# Novel T cell exhaustion gene signature to predict prognosis and immunotherapy response in thyroid carcinoma from integrated RNA-sequencing analysis

**DOI:** 10.1038/s41598-024-58419-7

**Published:** 2024-04-10

**Authors:** Yang Li, Zhen Wang, Fangting Lu, Yahu Miao, Qing Feng, Weixi Zhu, Qingqing Kang, Yijing Chen, Qiu Zhang

**Affiliations:** Department of Endocrinology, First Affiliated Hospital of Anhui Medical University, Hefei, China

**Keywords:** Thyroid carcinoma, T cell exhaustion, Tumor-associated macrophage, Immunotherapy, Single-cell RNA-sequencing, Gene signature, Cancer, Computational biology and bioinformatics, Genetics, Immunology

## Abstract

Exhausted CD8^+^ T lymphocytes and tumor-associated macrophages play critical roles in determining cancer prognosis and the efficacy of immunotherapy. Our study revealed a negative correlation between exhausted CD8^+^ T lymphocytes and prognosis in thyroid carcinoma (THCA). Consensus clustering divided patients into two subgroups of exhaustion with different prognoses, as defined by marker genes of exhausted CD8^+^ T cells. Subsequently, we constructed an eight-gene prognostic signature, and developed a risk score named the exhaustion-related gene score (ERGS) to forecast both prognosis and immunotherapy response in THCA. Bulk RNA sequencing analysis revealed a higher prevalence of M2 macrophages, indicative of an immunosuppressive tumor microenvironment (TME), in the high-ERGS group. Single-cell RNA sequencing showed that SPP1^+^ macrophages and CD14^+^ monocytes infiltrations were positively associated with higher ERGS. Functionally, it was determined that SPP1^+^ macrophages exert an immunosuppressive role, while CD14^+^ monocytes were implicated in promoting tumor progression and angiogenesis. Analysis of cell–cell interactions between SPP1^+^ macrophages and T cells highlighted the activation of the SPP1-CD44 and MIF-CD74 axes, both of which could foster an immunosuppressive TME. Therapeutic strategies that target SPP1^+^ macrophages, CD14^+^ monocytes, and the SPP1-CD44 and MIF-CD74 axes may potentially improve the prognosis and amplify the immunotherapy response in THCA patients.

## Introduction

Thyroid carcinoma (THCA) represents the most prevalent endocrine malignancy and ranks as the fifth leading cancer among women in the USA, exhibiting an increasing incidence worldwide^1^. It primarily consists of malignancies originating from follicular cells, notably including follicular thyroid carcinoma, invasive encapsulated follicular variant papillary carcinoma, and papillary thyroid carcinoma, as delineated by the latest 2022 WHO classification^2^. While the majority of patients respond to conventional therapies, including surgery, radioactive iodine treatment, and thyroid-stimulating hormone suppression, the efficacy of these approaches is limited for advanced or metastatic tumors that exhibit poor differentiation^3^. Recent clinical findings suggest that immunotherapy constitutes a promising targeted approach for treating advanced thyroid cancer^4^. Immunotherapy response is commonly predicted by *PD-L1* expression and tumor mutation burden (TMB) in melanoma and other cancers^5^. Nevertheless, THCA lacks sufficient biomarkers to predict the efficacy of immunotherapy. Thus, the identification of novel biomarkers and a predictive risk model for prognosis and immunotherapy response in THCA are crucial.

The tumor microenvironment (TME) is composed of tumor cells, stromal cells, immune cells, extracellular matrix components, and a plethora of cytokines. Emerging research underscores that the elements of the TME play a pivotal role in modulating tumor progression, patient prognosis, and the effectiveness of immunotherapy^6^. Host immune responses are vital in the clinical management of cancer, with CD8^+^ T lymphocytes recognized as the primary effector cells that penetrate tumors and mediate anticancer activities^7^. Within the TME, CD8^+^ T lymphocytes undergo relentless antigenic stimulation, attenuating their antitumor effectiveness. This state, known as "exhaustion," is chiefly characterized by the heightened expression of numerous immune checkpoint genes. The sustained activation of these inhibitory pathways can culminate in a state referred to as "terminal exhaustion"^7,8^. Immunotherapeutic strategies aim at these inhibitory receptors to rejuvenate the exhausted T cells, thereby enhancing antitumor responses^7,9^. Yet, the majority of patients derive limited benefit from immunotherapy, likely due to the immunosuppressive milieu of the TME^10^. Tumor-associated macrophages (TAMs) act as vital mediators, deemed indispensable in both tumor advancement and the modulation of antitumor immunity^10,11^. Hence, targeting TAMs amplifies the efficacy of immune checkpoint blockade (ICB) therapies by mitigating the immunosuppressive traits of the TME, a strategy validated in both experimental models and clinical trials^10–12^.

In this study, an eight-gene signature was established and validated based on the exhaustion-related genes (ERGs) to predict the prognosis and the immunotherapy response. By integrating the bulk RNA-sequencing (bulk RNA-seq) and single-cell RNA-sequencing (scRNA-seq) data, we further discussed the potential mechanisms and molecular targets of TAMs to improve immunotherapy response.

## Materials and methods

### Ethical statement

The data of the human tissue samples is de-identified and collected from the public databases. The approvement and informed consent can be found in the original article cited in this study. Therefore, no further approvement and consent are needed.

### Data acquisition

**Figure 1:**
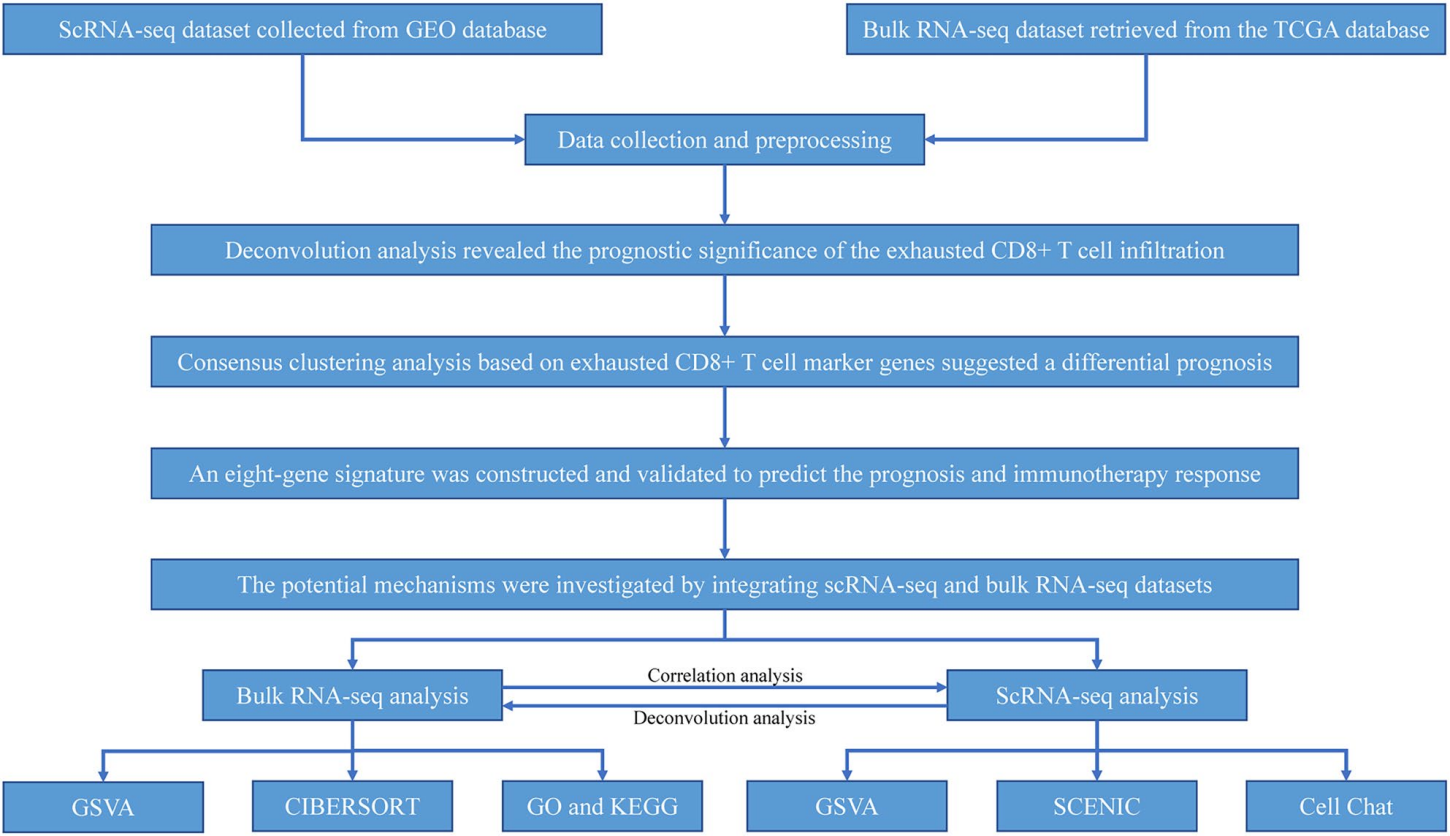
The workflow of the bioinformatic analysis. Datasets were collected from TCGA and GEO databases and preprocessed. Deconvolution analysis revealed the prognostic significance of the exhausted CD8^+^ T cell infiltration. Consensus clustering analysis based on exhausted CD8^+^ T cell marker genes suggested a differential prognosis. Therefore, an eight-gene signature was constructed and validated to predict the prognosis and immunotherapy response. The potential mechanisms were then investigated by integrating scRNA-seq and bulk RNA-seq datasets.

The schematic of our study's methodology is depicted in Fig. 1. A total of 819 samples were amassed for the study, comprising 498 thyroid cancer specimens harboring transcriptomic and clinical data from the TCGA database (Supplementary Table S1), 298 samples from patients treated with anti-PD-L1 from the IMvigor210 cohort (Supplementary Table S2), and 23 specimens featuring single-cell transcriptomic data of thyroid tumors from the Gene Expression Omnibus (GEO) database (Supplementary Table S3). Bulk RNA-seq count matrices were extracted from the TCGA database and converted to transcripts per million (TPM) formats for standardized cross-sample gene expression comparison. Transcriptomic data and clinical details of the IMvigor210 cohort, recipients of ICB therapy, were obtained from the public IMvigor210CoreBiologies package (http://research-pub.gene.com/IMvigor210CoreBiologies/)^13^. The 10× genomic scRNA-seq dataset (GSE184362) was sourced from the GEO database for in-depth analysis^14^.

### ScRNA-Seq data processing

All downstream scRNA-seq data processing was executed in R software employing the "Seurat" package (v4.1.1)^15^. Initially, cells were excluded if they exhibited any of the following: mitochondrial gene composition exceeding 10%, gene counts below 500, or above 5000. Additionally, genes related to mitochondria and ribosomes were removed. Next, the "DoubletFinder" (v2.0.3) was applied with its default settings to detect and remove potential doublets^16^. Following the filtration, a total of 164,852 cells remained for further analysis. After cell cycle correction, 5000 highly variable genes (HVGs) were chosen and subsequently scaled. The "Harmony" package (v0.1.0) was used to minimize batch effects^17^. Utilizing the top 20 principal components (PCs), cell clusters were delineated via the k-nearest neighbor algorithm at a 0.8 resolution. The t-distributed stochastic neighbor embedding (t-SNE) algorithm was employed to reduce the cell cluster features to two dimensions, clarifying the distinctions among clusters. Finally, differentially expressed genes (DEGs) were determined by the Wilcoxon test and Bonferroni correction and DEGs with a false discovery rate (FDR) < 0.01 and |log_2_ fold change (FC)|> 0.5 were chosen as candidates for cell annotation and marker genes. The second clustering replicated the first in methodology. For cell annotation, canonical markers from previous studies were selected^14,18,19^.

### Deconvolution of the scRNA-seq dataset

Deconvolution analyses of the scRNA-seq dataset were conducted to quantify cell infiltration levels in bulk RNA-seq samples, utilizing the 'BisqueRNA' package (v1.0.5)^20^. Considering the variable cellular composition across tissues, we isolated cells originating from primary tumors for subsequent examination and employed a reference-based approach to deconvolution—a method strongly advocated in the official documentation.

### Consensus clustering analysis

Initially, with the aid of the 'survival' package (v3.2), a univariate Cox regression analysis was performed to ascertain the prognostic significance of the cell-type composition within each bulk sample. Subsequently, patient stratification was executed using the 'ConsensusClusterPlus' package (v1.58), based on prognostic markers of exhausted CD8^+^ T cells^21^. Ultimately, comparisons of overall survival (OS) among the patient cohorts were drawn employing Kaplan–Meier survival curves and log-rank tests.

### Gene sets variation analysis (GSVA) and immune cell infiltration analysis

Pathway activities were calculated using the 'GSVA' package (v1.42), leveraging gene sets sourced from The Molecular Signatures Database^22^. Subsequent to the GSVA, variance analyses were conducted employing the 'limma' package (v3.50), leading to the identification of significant pathways (FDR < 0.01)^23^. Immune cell infiltration analysis was performed using the CIBERSORT algorithm, which discerns 22 immune cell types based on transcriptome data from bulk RNA-seq samples^24^. Furthermore, for single-cell level GSVA, gene sets drawn from prior studies were utilized^19^.

### Differentially expressed genes and functional enrichment analysis

To find the DEGs between two groups, we performed the DESeq2 algorithm using the "DESeq2" package (v1.34)^25^, and the genes with FDR < 0.05 and |log_2_ FC|> 2 were enrolled for subsequent analysis. Functional enrichment of the Gene Ontology (GO) and Kyoto Encyclopedia of Genes and Genomes (KEGG) pathways was employed utilizing the "clusterProfiler" package (v4.2.2) based on the latest online database (v7.4)^26^. Pathways with FDR < 0.01 were considered significant enrichment.

### Construction and verification of the risk model

Firstly, the TCGA dataset was randomly divided into training and testing sets for the purposes of model development and validation, respectively. Then, univariate Cox regression analysis was performed to assess the hazard ratios of the DEGs. Genes with FDR < 0.05 were regarded as prognostic. Subsequently, the least absolute shrinkage and selection operator (LASSO) algorithm was applied to minimize overfitting using the "glmnet" package (v4.1)^27^. We chose 1 − SE (standard error) as the tuning parameter, and with tenfold cross-validation, the best model was selected. Next, a stepwise multivariate Cox regression analysis was conducted to acquire coefficients based on the genes with non-zero coefficients. Finally, the risk score, which was named the exhaustion-related gene score (ERGS), was computed by multiplying the mRNA expression of the genes by their corresponding coefficients. Patients were then stratified into low-ERGS and high-ERGS cohorts based on the median value of ERGS. To evaluate prognostic significance, we applied the Kaplan–Meier survival curve and the log-rank test utilizing the "survminer" package (v0.4.9). With the help of the "timeROC" package (v0.4), we could generate a receiver operating characteristic (ROC) curve which displayed the precision of OS prediction at a given time point. Cox regression analysis was employed to estimate the hazard ratios for both ERGS and clinical features.

### Tumor mutation burden analysis

We downloaded the mutation profiles from the TCGA database. The "maftools" R package (v2.10) was then utilized to preprocess them in the form of mutation annotation format. Subsequently, the most frequently mutated genes were identified, and the TMB was computed. Finally, the frequency of mutated genes was compared across each ERGS group, and the relationship between ERGS and TMB was performed using correlation analysis.

### Prediction of immunotherapy response

Tumor Immune Dysfunction and Exclusion (TIDE, http://tide.dfci.harvard.edu/) was a website that could evaluate given transcriptomic data to predict immunotherapy efficacy^28^. T cell receptor (TCR) Shannon diversity index of the TCGA cohort, which has been demonstrated as a valuable biomarker of immunotherapy efficacy, was collected from the previous study^29^. By combining the transcriptomic data and the immunotherapy response of 298 bladder cancer patients from the IMvigor210 cohort, the prediction of the immunotherapy response of the ERGS was validated^13^. ERGS for each patient was calculated as detailed earlier, with the median ERGS serving to split patients into distinct categories.

### Single-Cell regulatory network inference and clustering (SCENIC) analysis

SCENIC analysis was extensively utilized to determine the gene regulatory network in many cell types using scRNA-seq data^30^. PySCENIC (v0.12.0), a faster version of the SCENIC analysis, was implemented according to the pipeline provided in the official tutorial. All the associated databases can be downloaded from the public website (https://resources.aertslab.org/cistarget/).

### Cell–cell communication analysis

To discover the underlying cell–cell communications, the "CellChat" package (v1.4.0) was performed according to the official tutorial^31^. The databases of secreted signaling, ECM-receptors, and cell–cell contact, were obtained from the "CellChat" package. The noteworthy interactions between specific cell types were chosen for analysis and visualization.

### Statistical analysis

Statistical analyses were conducted using R (v4.1.2) and Python (v3.9.12). For comparing two groups, the Wilcoxon rank-sum test was employed. The Kruskal–Wallis test was utilized for comparisons across multiple groups. Spearman correlation analysis was conducted to assess the linear relationship between two continuous variables. The Benjamini–Hochberg method was applied to calculate the multiplicity-corrected p-value, known as the FDR, to identify DEGs. All tests were two-tailed, and a p-value lower than 0.05 was considered statistically significant.

## Results

### Identification of exhausted CD8^+^ T cell and its marker genes

**Figure 2:**
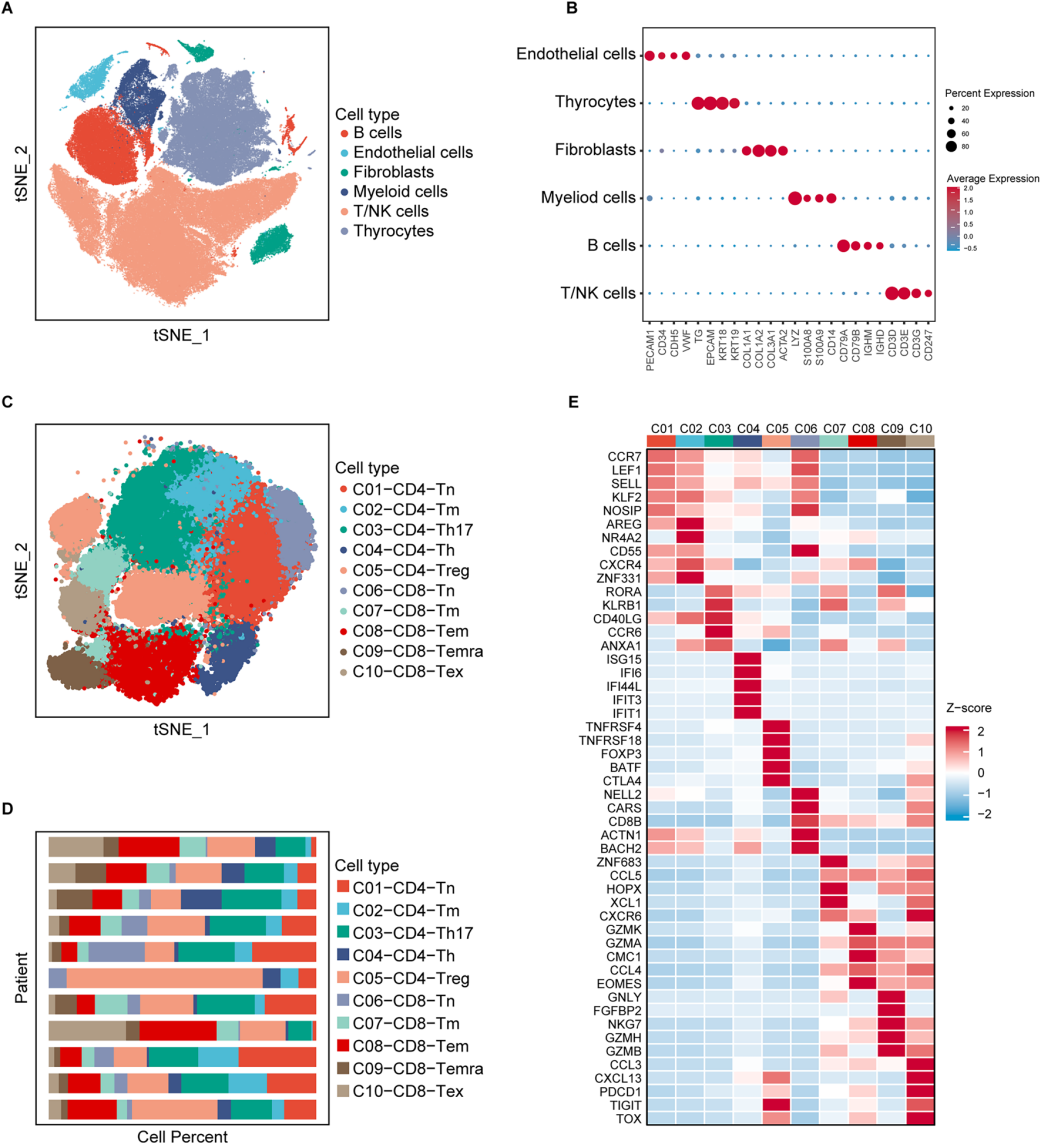
Single-cell RNA-sequencing analysis identified exhausted CD8^+^ T cell marker genes. (**A**) t-SNE plot showed six main cell types. (**B**) Dot plot displayed the canonical markers of the six main cell types. (**C**) t-SNE plot colored by T cell subtypes. (**D**) The proportion of T cell subtypes in each scRNA-seq sample. (**E**) Heatmap showed the expression of classical marker genes of the T cell subtypes.

After data processing (Supplementary Fig. S1), a total of 164,852 cells from 7 primary tumors, 6 para-tumor tissues, 8 metastatic lymph nodes, and 2 distant metastases were incorporated into further analysis. For the first round of reduction and clustering, six primary cell subtypes were identified, including T/natural killer (NK) cells, B cells, myeloid cells, fibroblasts, thyrocytes, and endothelial cells (Supplementary Fig. S2; Fig. 2A,B). Subsequently, we performed further reduction on T/NK cells, and finally, ten main cell populations (Fig. 2C; Supplementary Fig. S3) from different tissues of different patients (Fig. 2D) were identified based on the DEGs of each cell cluster (Supplementary Table S4). Each cell cluster was annotated mainly according to its functional molecule and canonical markers (Fig. 2E)^18^. For example, the C10-CD8-Tex cluster was characterized by upregulated genes of immune checkpoints (*PDCD1, HAVCR2, LAG3,* and *TIGIT*) and associated transcription factors (*EOMES, TOX,* and *RBPJ*), which corresponded to exhausted CD8^+^ T cells.

### The transcriptome characteristics of the consensus clustering based on the exhausted CD8^+^ T cell marker genes

**Figure 3:**
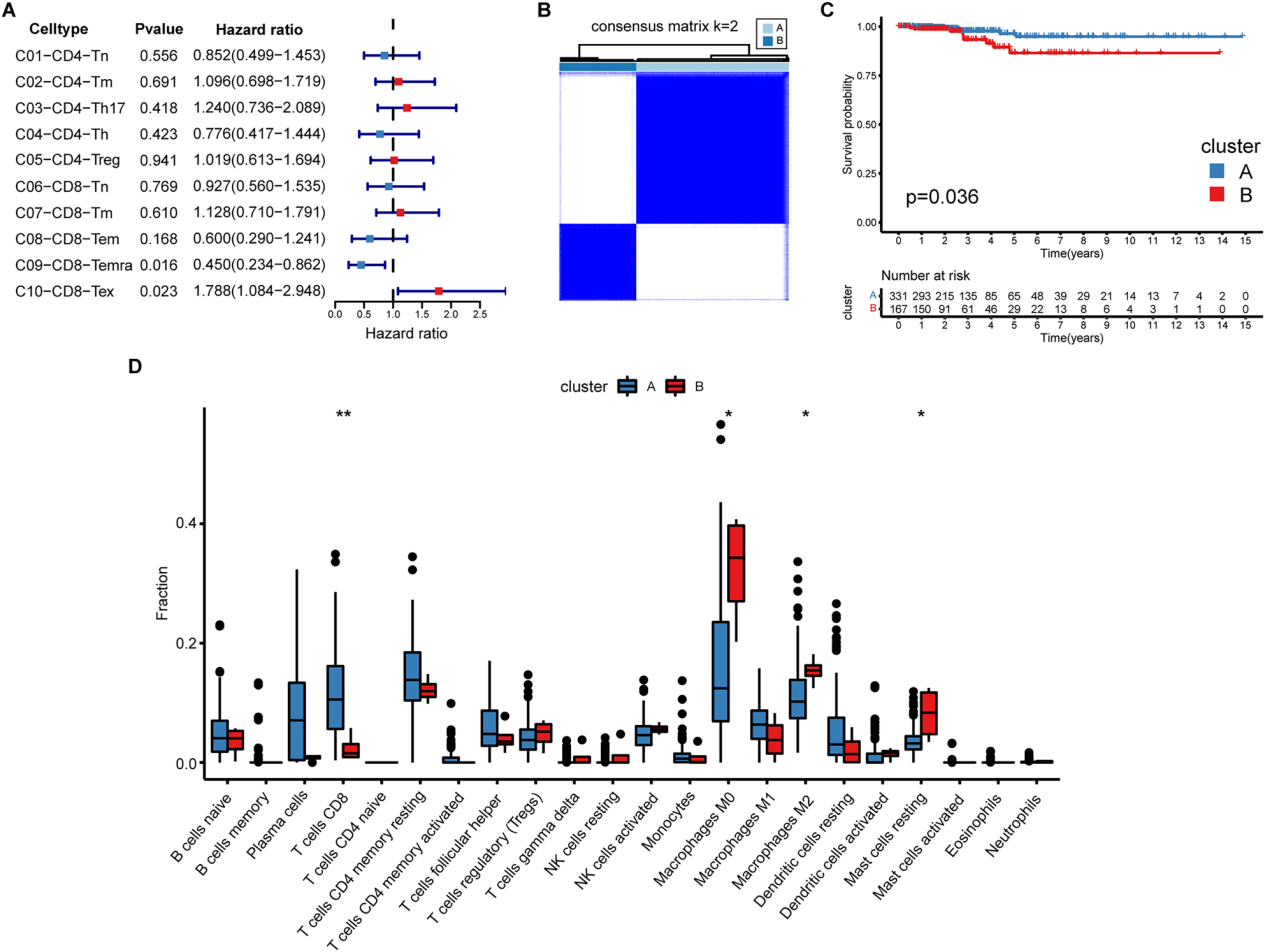
The transcriptomic features of the consensus clustering based on the exhausted CD8^+^ T cell marker genes. (**A**) Forest plot showed the prognostic value of the T cell subtypes utilizing the deconvolution analysis. (**B**) Two exhaustion subtypes were identified using consensus clustering based on the exhausted CD8^+^ T cell marker genes. (**C**) The comparison of the prognosis between the two exhaustion states using the Kaplan–Meier survival curve and the log-rank test. (**D**) The comparison of immune cell infiltrations between the two clusters using the CIBERSORT algorithm.

To assess T cell clusters' prognostic significance, we conducted a univariate Cox regression by utilizing bulk RNA-seq deconvolution (Supplementary Table S5). We discovered the C10-CD8-Tex was negatively associated with patients' OS (p = 0.023) (Fig. 3A). Consensus clustering was carried out based on the C10-CD8-Tex prognostic marker genes. Next, two exhaustion-related subtypes have been identified (Fig. 3B), characterized by distinct expressions of immune checkpoint genes (Supplementary Fig. S4A). A significant difference in the prognosis has been observed (Fig. 3C). For pathway analysis (Supplementary Fig. S4B), we found a comprehensive downregulation of immune-related pathways in the worse prognosis cluster. The GO and KEGG enrichment analyses also showed the same results (Supplementary Fig. S4C,D). The immune cell infiltration analysis (Fig. 3D) demonstrated that CD8^+^ T lymphocyte infiltration decreased while M2 macrophage increased within the worse prognosis cluster.

### Establishment and validation of the eight-gene prognostic signature based on the ERGs

**Figure 4:**
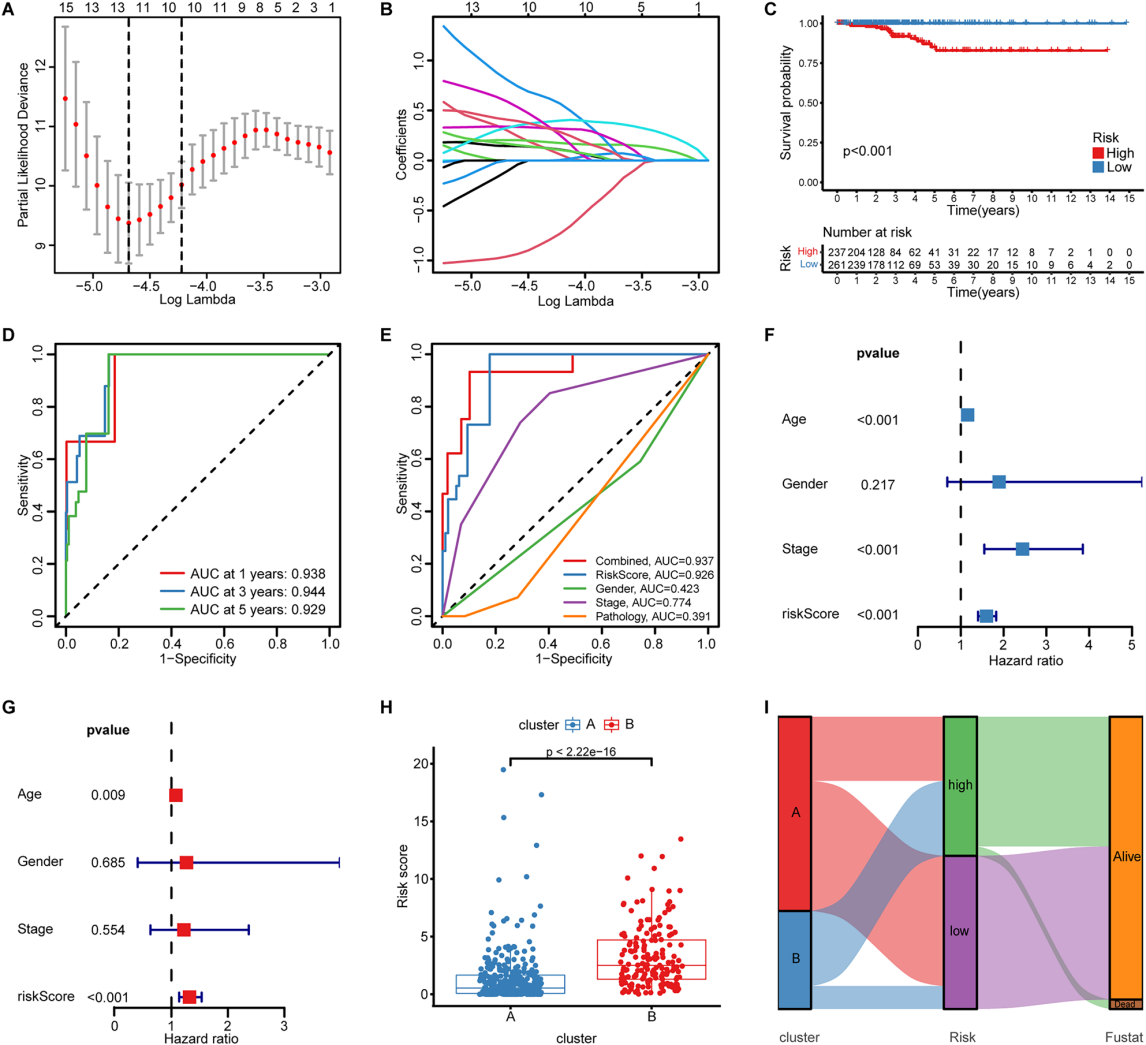
The construction of an eight-gene prognostic signature based on exhaustion-related genes. (**A**) Penalty plot of the LASSO analysis of the 15 prognostic exhaustion-related genes. (**B**) Variation of the gene coefficient with an increasing penalty parameter. (**C**) The Kaplan–Meier survival curve and the log-rank test showed an inferior prognosis in the high-ERGS group. (**D**) AUC curves showed the accuracy of 1-, 3-, 5-year OS prediction in the TCGA THCA cohort. (**E**) The ERGS had the highest prediction accuracy compared to other clinical features. (**F**,**G**) Forest plots of univariate and multivariate Cox regression analysis indicated an independent risk factor. (**H**) Boxplot showed that the cluster with a worse prognosis had a higher ERGS. (**I**) Sankey plot displayed the relationship of the consensus clusters, ERGS groups, and survival states.

To find the DEGs between the two exhaustion subtypes, we applied the DESeq2 algorithm, and 459 genes with FDR < 0.05 and |log_2_ FC|> 2, which were named ERGs, were analyzed by univariate Cox regression (Supplementary Table S6). Next, 70 prognostic genes (p < 0.05) were utilized for further investigation (Supplementary Table S7). The TCGA cohort was randomly split into a training set and a test set, both of which had similar clinical characteristics (Supplementary Table S8). The TCGA training cohort was used for model construction, and the test set was used for model validation. After LASSO Cox regression analysis, 12 genes with non-zero LASSO coefficients (Fig. 4A,B) were used for stepwise multivariate Cox regression analysis, and finally, eight genes (*CXCL9, CYP17A1, DRGX, ENTHD1, HAS1, LAIR2, RETN,* and *SPHKAP*) were screened out for the construction of the risk model (Supplementary Table S9). The TCGA training set was divided into a high-ERGS (n = 124) and low-ERGS (n = 125) group according to the median ERGS in the training set, and the test set was likewise classified into a high-ERGS (n = 113) and low-ERGS (n = 136) group (Supplementary Table S10).

We discovered that patients older than 50 or who were male seemed to have a higher ERGS (Supplementary Fig. S5A,B). However, other clinical features showed no statistical significance (Supplementary Fig. S5C–F). We also found that the high-ERGS group obtained an inferior prognosis compared to the low-ERGS group in the training, test (Supplementary Fig. S6A,B), and all cohorts (Fig. 4C). Next, we conducted the time-dependent ROC curves, and the 1-, 3-, and 5-year AUC values were 0.938, 0.944, and 0.929, respectively (Fig. 4D). The training and test sets also showed satisfying AUC values (Supplementary Fig. S6C,D). Meanwhile, the ERGS showed the highest AUC values compared with other clinical features (Fig. 4E), suggesting a powerful prognostic signature. Furthermore, the accuracy of OS prediction was strengthened when integrating the ERGS and clinical features (Fig. 4E). Cox regression analyses also indicated that the ERGS was an independent risk factor (p < 0.001, hazard ratio = 1.321, 95% CI 1.137–1.535) (Fig. 4F,G). The ERGS distribution was statistically different between the two exhaustion subtypes (Fig. 4H). The Sankey diagram (Fig. 4I) also suggested a close relationship between exhaustion subtype, ERGS group, and survival state.

### The eight-gene signature could predict immunotherapy response in THCA patients

**Figure 5:**
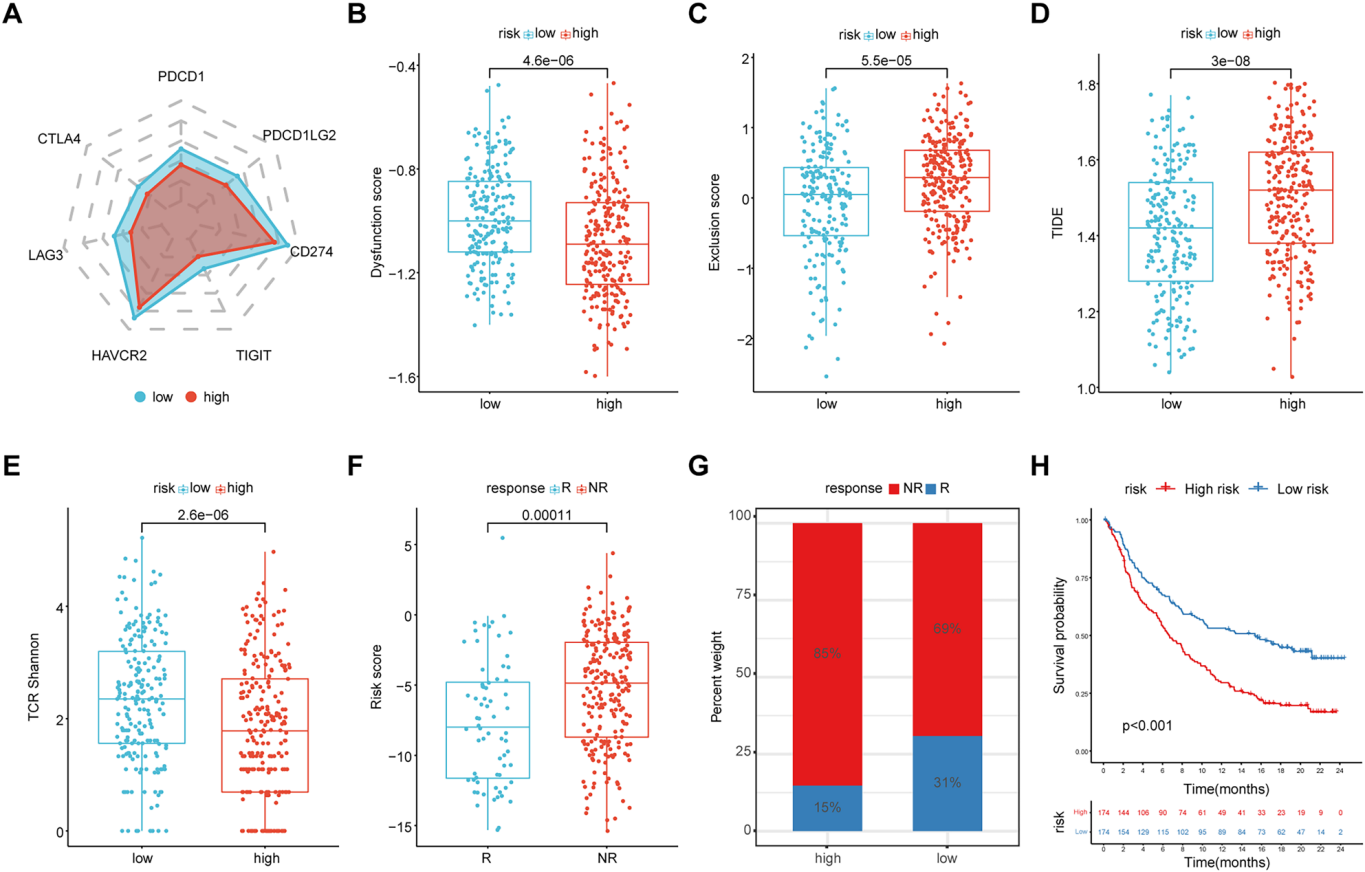
The eight-gene signature could predict immunotherapy response in THCA patients. (**A**) The expression of immune checkpoint genes in each ERGS group. (**B–E**) The dysfunction score, exclusion score, TIDE score, and TCR Shannon diversities of the high-ERGS and low-ERGS groups. (**F**) Patients who did not respond to immunotherapy had a higher ERGS. (**G**) Patients in the high-ERGS group were less likely to benefit from immunotherapy. (**H**) Patients classified into the high-ERGS group had an inferior prognosis, although they have received immunotherapy.

First, we compared the gene expression of well-known immunotherapy biomarkers between each ERGS group and discovered a significant downregulation of immune checkpoint genes in the high-ERGS group (Fig. 5A). Next, the TIDE website tools showed that the high-ERGS group had a lower dysfunction score but a higher exclusion score and TIDE score (Fig. 5B–D; Supplementary Table S11), indicating a less beneficial treatment of the ICBs. Furthermore, the TCR Shannon diversity index also suggested that tumors with high-ERGS had a lower TCR Shannon diversity index (Fig. 5E; Supplementary Table S11) and could be insensitive to the ICBs treatment. Finally, patients who underwent anti-PD-L1 therapy in the IMvigor210 cohort were utilized to evaluate the prediction effectiveness of the ERGS (Supplementary Table S12). We found that higher ERGS was associated with worse anti-PD-L1 effectiveness (p = 0.00011; Fig. 5F), and the treatment efficacy was decreased in the high-ERGS group (p = 0.0015; Fig. 5G). Survival analysis suggested an inferior prognosis after anti-PD-L1 treatment in the high-ERGS group (p < 0.001; Fig. 5H).

### Tumors in the high-ERGS group have an immunosuppressive TME with higher M2 macrophage infiltration but lower M1 macrophage and CD8^+^ T cell infiltration

**Figure 6:**
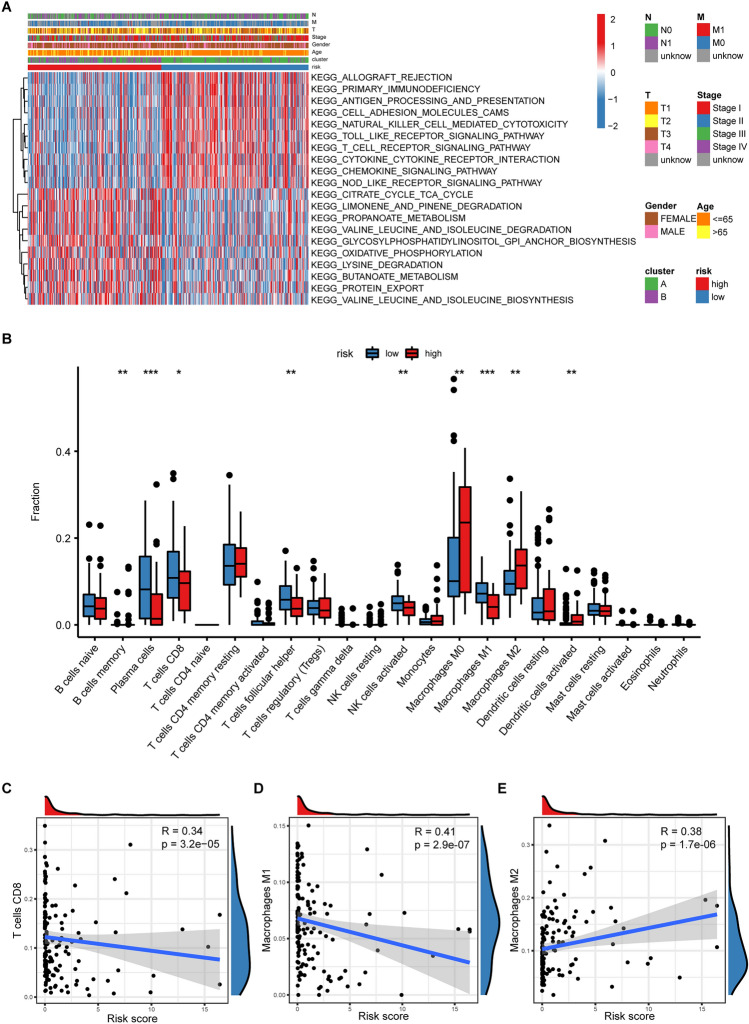
The transcriptome features of the ERGS group. (**A**) Heatmap showed the top 10 upregulated KEGG pathways in the low-ERGS and high-ERGS groups with corresponding clinical characteristics. (**B**) The comparison of immune cell infiltrations between the high-ERGS and low-ERGS groups calculated by the CIBERSORT algorithm. (**C–E**) The correlation analysis demonstrated a positive linear correlation between M2 macrophage infiltration and ERGS but a negative linear correlation between M1 macrophage or CD8^+^ T cell infiltration and ERGS.

First, we discovered that numerous immune-related pathways were downregulated in the high-ERGS group (Fig. 6A), suggesting an immunosuppressive TME with impaired function of antigen presentation, innate immunity, and cytokine or chemokine production. Meanwhile, there was also a comprehensive upregulation of metabolic pathways in the high-ERGS group. Furthermore, the GO and KEGG analyses also suggested similar results (Supplementary Fig. S7). Subsequently, CIBERSORT results demonstrated that the high-ERGS tumors had decreased infiltrations of CD8^+^ T cells and M1 macrophages but increased infiltrations of M2 macrophages (Fig. 6B). Hopefully, the ERGS was strongly correlated with immune cell infiltration. For instance, the CD8^+^ T cell and M1 macrophage infiltrations showed a negative linear correlation with the ERGS (Fig. 6C,D ). However, the M2 macrophage showed a positive linear correlation (Fig. 6E). These results further confirmed the immunosuppressive nature of the TME in the high-ERGS group.

The relationship between somatic mutations and ERGS was then explored. First, the top 10 genes with the highest frequency of mutations in each ERGS group were identified (Supplementary Fig. S8A,B). Although they had similar mutated genes, we found that BRAF (p = 0.0012) and NRAS (p = 0.011) were considered statistically significant between the two groups (Supplementary Fig. S8C,D). Next, the TMB was calculated, suggesting that the high-ERGS tumors had increased TMB (Supplementary Fig. S8E). Moreover, the ERGS was shown to be positively correlated with TMB (p = 0.0015, R = 0.15) (Supplementary Fig. S8F).

### SPP1^+^ macrophage and CD14^+^ monocyte infiltrations were correlated with ERGS and associated with M2 macrophage infiltration and angiogenesis

As mentioned, the high-ERGS group had an immunosuppressive TME, and the ERGS was highly correlated with M1 and M2 macrophage infiltrations. Therefore, we concentrated on the myeloid cells in the TME to better explain the underlying biological processes.

**Figure 7:**
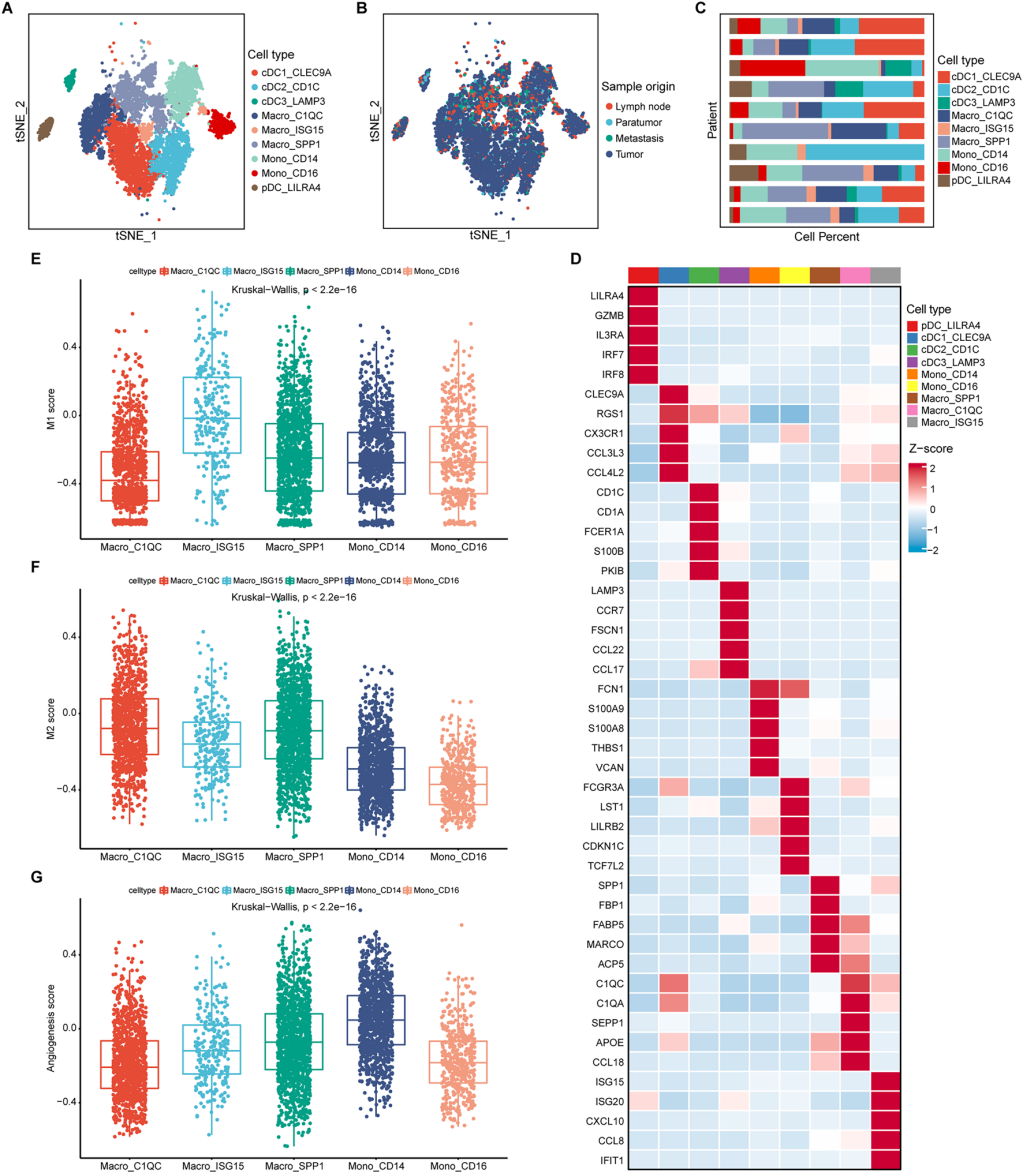
The identification of myeloid cell subtypes. (**A**,**B**) t-SNE plots showed the nine myeloid cell subtypes and their tissue distributions. (**C**) Bar plot displayed the proportion of myeloid cell subtypes in each patient. (**D**) Heatmap showed the expression of canonical marker genes of myeloid cell subtypes. (**E**–**G**) The GSVA results of the classical gene sets of M1 macrophage, M2 macrophage, and angiogenesis.

The myeloid cells of 23 thyroid carcinoma samples collected from GSE184362 were extracted for further reduction, clustering, and annotation (Supplementary Fig. S9). According to the previous pan-cancer study^19^, nine cell types were annotated (Fig. 7A), which came from different tissues or patients (Fig. 7B,C). For instance, cells that specifically expressed *SPP1*, *FBP1*, *FABP5*, *MARCO*, and *ACP5* were annotated as SPP1^+^ macrophages (Fig. 7D). Subsequently, to describe the biological functions of each cell type, we applied the GSVA to compute the canonical makers of TAMs^19^. We discovered that the ISG15^+^ macrophage had the highest M1 score (p < 2.2e−16; Fig. 7E), indicating an M1-like macrophage. However, the SPP1^+^ macrophage showed the highest M2 score (p < 2.2e−16; Fig. 7F), suggesting the anti-inflammatory and pro-tumor characteristics of M2-like macrophages. Furthermore, the CD14^+^ monocyte with the highest angiogenesis score played a crucial role in tumor progression (p < 2.2e−16; Fig. 7G). Next, the infiltration of each cell type was calculated using the deconvolution of the scRNA-seq data (Supplementary Table S13). CD14^+^ monocyte infiltration was identified as a risk factor using Cox regression analysis and the survival analysis also showed a negative association with the OS of THCA patients (Supplementary Fig. S10A,B). However, limited evidence was found to support the poor prognosis of SPP1^+^ macrophage infiltration (Supplementary Fig. S10A,C). Moreover, the infiltration of the SPP1^+^ macrophage and the CD14^+^ monocyte positively correlated with the ERGS (Supplementary Fig. S10D,E), which deserved further investigation.

### The transcriptome features of the CD14^+^ monocyte and SPP1^+^ macrophage

To explore the function of the cell, we conducted the GSVA algorithm using the gene sets of KEGG pathways. For SPP1^+^ macrophages, there was a comprehensive upregulation of several metabolism-related pathways (Supplementary Fig. S11A). Higher glutathione levels in the TME are related to tumor development, enhanced metastasis, and resistance to chemotherapy and radiotherapy^32^. Meanwhile, the PPAR family is critical to M1/M2 macrophage polarization and serves as a key regulator of oxidative phosphorylation and energy homeostasis in macrophages^33,34^. Furthermore, the activities of glycolysis, gluconeogenesis, oxidative phosphorylation, and the TCA cycle, which are considered significant for M2 macrophages, are higher than those of other myeloid cells^35^. For CD14^+^ monocytes, there was also substantial upregulation of tumor-associated pathways (Supplementary Fig. S11A). The higher activity of the bladder cancer pathway demonstrates a higher probability of invasion and metastases^36^. Likewise, the VEGF signaling pathway is associated with angiogenesis and could contribute to tumor metastasis^37^. Moreover, the ErbB signaling pathway is correlated with tumorigenesis, immune escape, and resistance to immunotherapy^38^. In addition, the TGF-beta signaling pathway could also promote tumorigenesis and metastasis^39^.

The SCENIC analysis revealed the potential gene regulatory network of each cell type. For SPP1^+^ macrophages, the top 5 TFs with the highest activity were ATF7, PPARG, TFDP1, HMGA1, and NFIB (Supplementary Fig. S11B). ATF7 inhibits the expression of innate immunity-related genes in macrophages^40^. PPARG is essential to the polarization of M2 macrophages and functions as a critical regulator of cell metabolism^33,34^. HMGA1 was found to affect cell proliferation, apoptosis, and autophagy, which were linked to tumor progression^41^. For CD14^+^ monocytes, the top 5 TFs were ZBTB7B, FOSL2, KLF8, FOSB, and CLOCK (Supplementary Fig. S11B). FOSL2 activation in macrophages promotes the polarization of M2 macrophages and is related to lung cancer development and migration^42^. Overexpressed KLF8 in cancer cells contributes to tumor metastasis through extracellular matrix remodeling and increased angiogenesis^43^.

### The landscape of SPP1^+^ macrophage and CD14^+^ monocyte crosstalk with T cell

It was found that SPP1^+^ macrophages and CD14^+^ monocytes widely communicated with T cells based on the CellChat results (Supplementary Fig. S12A). There were several interactions in common. The interactions between the MHC molecules and CD4/CD8 receptors represent the antigen presentation activity of macrophages. CCL3 exerts both antitumor and pro-tumorigenic effects by recruiting cytotoxic CD8^+^ T cells or Tregs. The interactions of CLEC2B-KLRB1 and CLEC2C-KLRB1 induce T cell activation^44^. LGALS9 could induce immune cell death and the differentiation and maintenance of Tregs^45,46^.

SPP1^+^ macrophages had some unique interactions (Supplementary Fig. S12A). The interaction of SPP1-CD44 inhibits T-cell activation and promotes tumor immune evasion^47,48^. The MIF-CD74 interaction could suppress both CD8^+^ T cell infiltration and pro-inflammatory M1 conversion of macrophages in the TME^49^. For CD14^+^ monocytes, the extracellular NAMPT contributes to tumor angiogenesis, decreased antitumor immunity, and resistance to ICBs^50,51^. THBS1 could affect the immune cell by inducing senescence or cell death via the CD47 receptor^52^.

### The landscape of SPP1^+^ macrophage and CD14^+^ monocyte crosstalk with tumor cell

Several shared cell–cell interactions were critical to tumor development (Supplementary Fig. S12B). Secreted NAMPT in the TME promotes tumor proliferation by increasing the NAD^+^ pool and affects cancer metastasis and treatment resistance in many solid human tumors^50,51^. The interaction of HBEGF-EGFR increases tumor cell intravasation and metastasis^53^. GRN stimulated tumor cell proliferation, migration, and invasion^54^.

However, both SPP1^+^ macrophages and CD14^+^ monocytes had many unique communications (Supplementary Fig. S12B). For SPP1^+^ macrophages, the activation of the SPP1-CD44 axis promotes the stemness of the tumor cells, contributing to tumor metastasis in pancreatic cancer^48^. For CD14^+^ monocytes, the VEGFA/PGF-VEGFR1 axes play a significant role in tumor angiogenesis, which is related to a poorer prognosis^37,55^. The EREG/AREG-EGFR communications serve as a pivotal factor in tumor proliferation and migration in several solid human cancers^56^.

## Discussion

In this study, we established an eight-gene signature collected from the ERGs to predict prognosis and immunotherapy response in THCA. Moreover, we further discussed the potential molecular mechanisms and targets which might benefit the immunotherapy response.

Utilizing the deconvolution analysis, exhausted CD8^+^ T lymphocytes were found to serve as a risk factor that attracted our attention. Two exhaustion subtypes with different prognosis were clustered based on the marker genes of exhausted CD8^+^ T lymphocytes. To explore the distinct biological process in the two exhaustion states, we performed differential expression analysis, and the DEGs were called ERGs, from which an eight-gene model was constructed and the ERGS was computed. Patients with a higher ERGS have an inferior prognosis than those with a lower ERGS, and the ERGS can effectively predict the prognosis of THCA patients compared with other clinical features. Furthermore, Cox regression analyses demonstrated that the ERGS served as an independent risk factor. For transcriptomic features, GSVA results revealed that the high-ERGS group had an immunosuppressive TME and abnormal metabolic pathways. Moreover, the high-ERGS tumors had increased infiltrations of M2 macrophage but decreased infiltrations of M1 macrophage and CD8^+^ T cell. Meanwhile, the ERGS had a positive linear correlation with M2 macrophage infiltration but a negative linear correlation with M1 macrophage and CD8^+^ T cell infiltrations. All these features indicate that tumors with high ERGS have an immunosuppressive TME due to the infiltration of M2 macrophages and the lack of M1 macrophages and CD8^+^ T cell, which could be the leading contributor to the poor prognosis.

The discrepancy in immune cell infiltration and function across ERGS groups encouraged us to investigate the predictive efficacy of ERGS for immunotherapy response. We first analyzed the expression of 7 immune checkpoint genes and found they were increased in the low-ERGS group. Next, the low-ERGS tumors had higher TCR diversities. Subsequently, TIDE analysis suggested patients with low ERGS were more likely to benefit from immunotherapy due to their lower TIDE score^28^. To further verify the predictive value of ERGS, an immunotherapy cohort (the Imvigor210 cohort) was used for validation, despite the fact that it is a bladder cancer cohort^13^. Hopefully, we discovered that patients in this cohort with reduced ERGS obtained a greater immunotherapy response rate and a longer survival time. Taken together, we can infer that THCA patients with lower ERGS may have higher probabilities of benefiting from immunotherapy.

Considering the macrophage infiltrations and immunosuppressive TME between different ERGS groups, we focused on the TAMs for further investigation. We discovered that the SPP1^+^ macrophage was more likely to be M2-like, and the CD14^+^ monocyte could contribute to tumor angiogenesis. According to the CellChat results, several vital communications in common were found. For SPP1^+^ macrophages, SPP1-CD44 inhibits T-cell activation and promotes tumor immune evasion^47,48^. The MIF-CD74 interaction could suppress both CD8^+^ T cell infiltration and pro-inflammatory M1 conversion of macrophages in the TME^49^. For CD14^+^ monocytes, the VEGFA/PGF-VEGFR1 axes play a significant role in tumor angiogenesis^37,55^, and the EREG/AREG-EGFR communications serve as a critical factor in tumor proliferation in several solid human cancers^56^. In conclusion, we revealed that SPP1^+^ macrophages and CD14^+^ monocytes were highly associated with immunosuppressive TME and tumor angiogenesis, respectively.

In this research, the ERGS was composed of 8 ERGs (*CXCL9, CYP17A1, DRGX, ENTHD1, HAS1, LAIR2, RETN,* and *SPHKAP*), some of which were pivotal to the tumor progression. CXCL9 is located in the dendritic cells and macrophages and could generate a "hot" TME^57^. CYP17A1, a key enzyme for producing several steroids, is considered a risk factor for prostate cancer^58^. HAS1 encodes hyaluronic acid, and its upregulation potentiates tumor development and progression by remodeling the TME^59^. LAIR2 is proven to be a T cell exhaustion biomarker and correlated with worse survival in cholangiocarcinoma^60^. RETN has a significant impact on tumor growth, metastasis, angiogenesis, and therapy resistance^61^.

TAMs represent a critical category of immune cells within the TME, playing multifaceted roles in tumor growth, metastasis, immune evasion, and therapeutic response. The significant function of SPP1^+^ macrophages across various cancer contexts, especially in terms of tumor progression, metastasis, and the modulation of immune responses, is noteworthy. SPP1, a protein secreted by macrophages, plays a pivotal role in cellular communication and immune regulation within the TME. One study demonstrated that the SPP1^+^ macrophage subpopulation promotes endothelial invasion and metastasis of tumor cells in the TME of head and neck squamous cell carcinoma^62^. Additionally, research in colorectal cancer found that SPP1^+^ macrophages are associated with suppressed T cell infiltration in the TME, with a higher presence of SPP1^+^ macrophages correlating with less benefit from immunotherapy^63^. CD14^+^ monocytes are crucial in regulating the tumor's immune response, facilitating tumor proliferation and metastasis. In high-grade serous ovarian cancer, CD14^+^ monocytes play a key regulatory role in tumor progression, including the modulation of tumor inflammation and angiogenesis^64^. Furthermore, CD14^+^ monocytes can suppress the action of T cells, associated with poor outcomes in melanoma patients^65^. This comprehensive analysis underscores the complex interplay between different immune cell types within the TME, highlighting their potential impact on cancer progression and treatment response.

Although this study obtained promising findings, it also had several limitations. Firstly, the eight-gene signature was constructed and validated only on the TCGA THCA cohort. Thus, more thyroid cancer datasets need to be utilized to further validate the prognostic value of our ERGS in the future. Secondly, further transcriptome-level and protein-level verification is required to demonstrate the differential expression between the normal and tumor tissues. Absolutely, in vitro and in vivo experiments are needed to confirm the potential mechanisms postulated by us. Lastly, the predictive ability of immunotherapy response is limited due to the lack of immunotherapy cohorts in THCA, and the bioinformatic analyses could only serve as a suggestion. The target cells and axes will need experimental and clinical verification in the future.

In conclusion, we established and validated an eight-gene signature based on the ERGs which could predict prognosis and immunotherapy response in THCA patients. Furthermore, SPP1^+^ macrophage was revealed to have an immunosuppressive function, and CD14^+^ monocyte was found to contribute to tumor progression and angiogenesis. Finally, the SPP1-CD44 and MIF-CD74 axes in the communication between SPP1^+^ macrophages and T cells might be candidates for target therapy, which might reverse the immunosuppressive TME and enable patients to benefit from immunotherapy.

### Supplementary Information

Supplementary Information.

## Data Availability

The datasets used and provided here are available for download from various digital archives. Both the repository(s) and accession number(s) are listed in the article/supplementary materials.
