# Supplementary material for: Novel T cell exhaustion gene signature to predict prognosis and immunotherapy response in thyroid carcinoma from integrated RNA-sequencing analysis
Source: Sci Rep. 2024 Apr 10;14:8375. doi: 10.1038/s41598-024-58419-7 (PMC11006682; doi:10.1038/s41598-024-58419-7)
Supplement: Supplementary file 1 — Supplementary Information. [file 41598_2024_58419_MOESM1_ESM.zip › Supplementary Figures.DOCX]

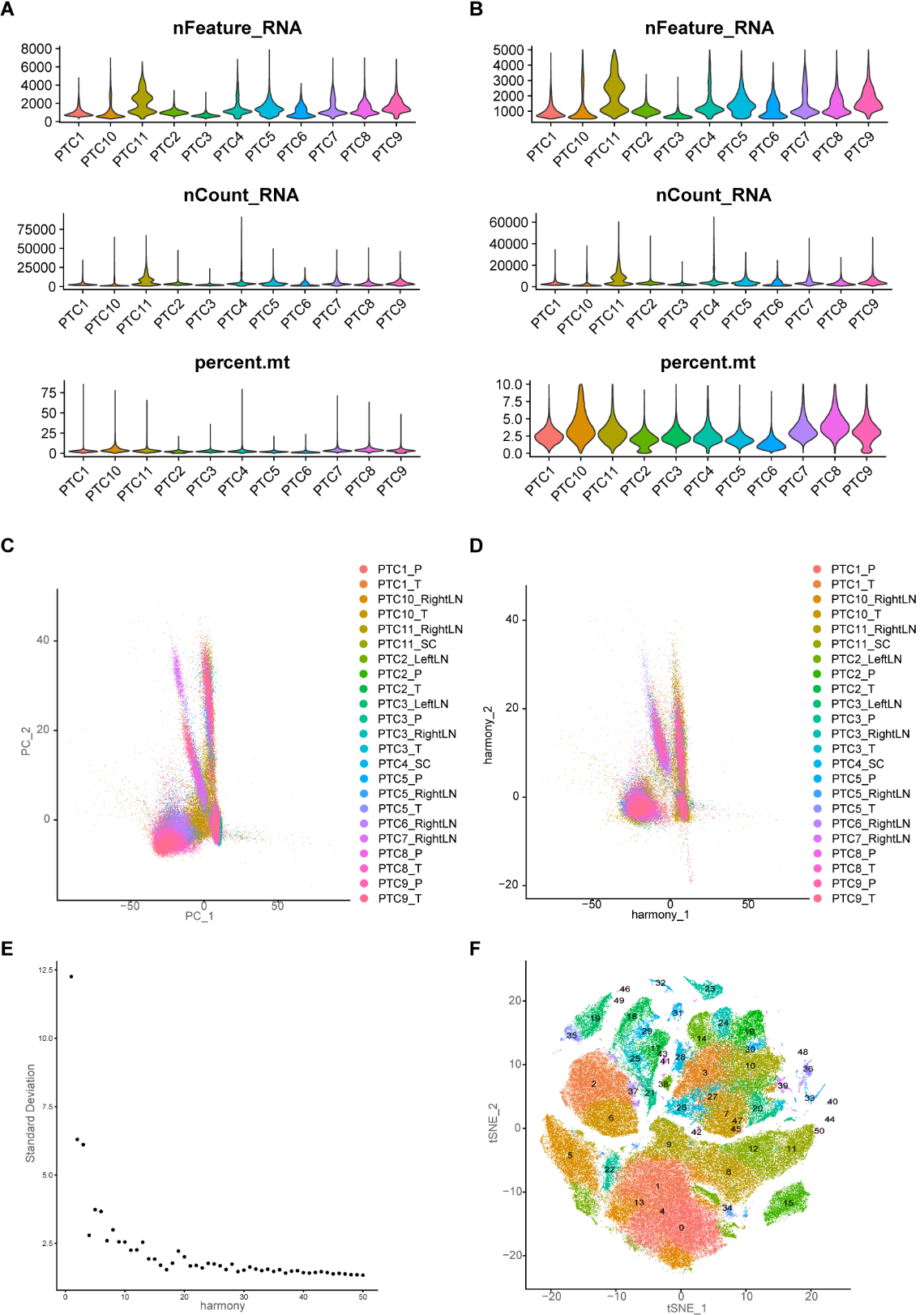


**Supplementary Figure S1.** The quality control and preprocessing of the scRNA-seq dataset. **(A), (B)** The expression of the RNA feature, RNA count, and percentage of the mitochondrial gene before and after quality control. **(C), (D)** The batch effect before and after the Harmony processing. **(E)** The elbow plot showed the ideal selection of the principal component. **(F)** t-SNE plot displayed the 50 subclusters after the "Seurat" pipeline.


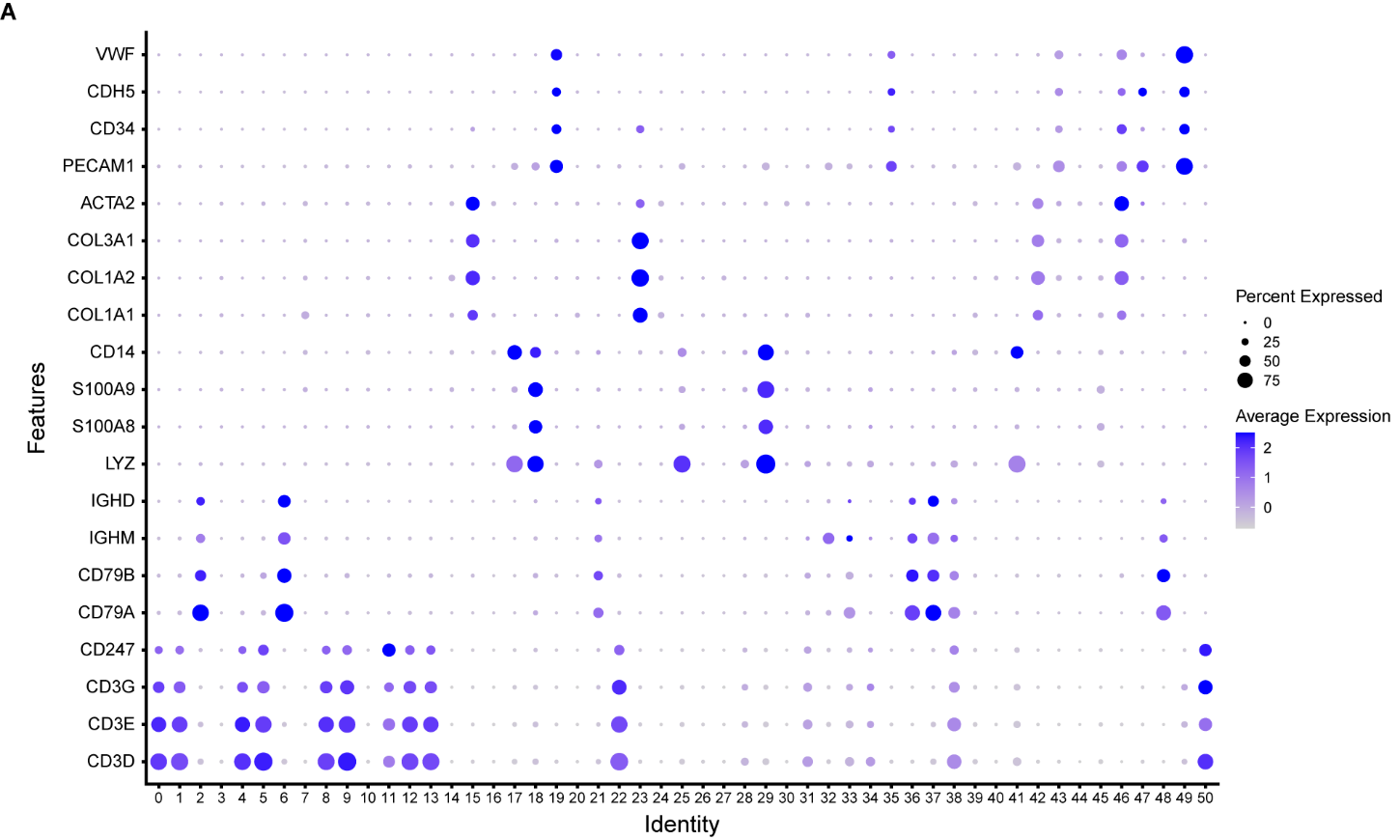


**Supplementary Figure S2.** The identification of the six main cell types. **(A)** The dot plot showed the expression of the marker genes in the 50 subclusters.


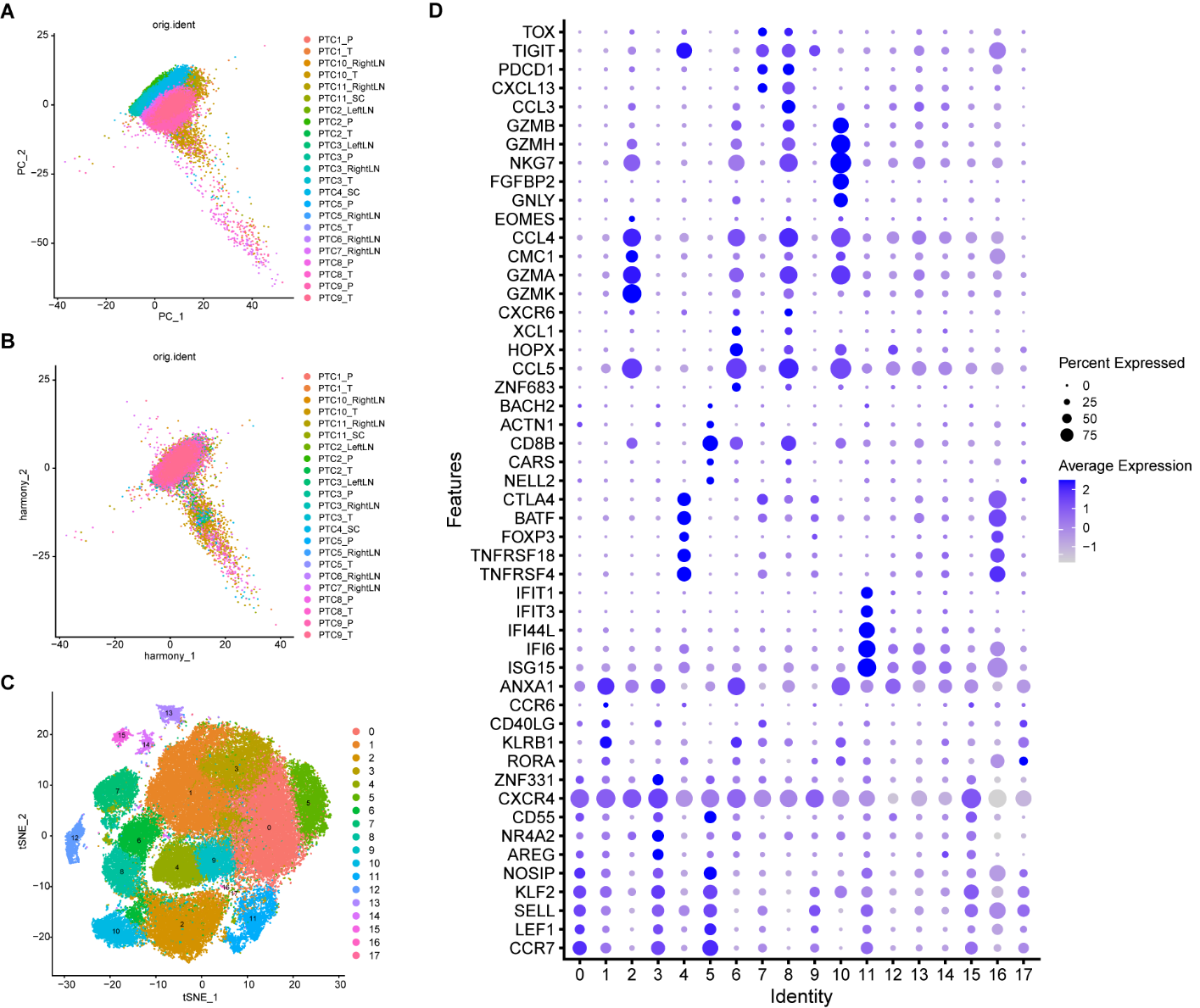


**Supplementary Figure S3.** The identification of the T cell subtypes. **(A), (B)** The batch effect before and after the Harmony processing. **(C)** t-SNE plot displayed the 17 subclusters after the "Seurat" pipeline. **(D)** The dot plot showed the expression of the marker genes in the 17 subclusters.


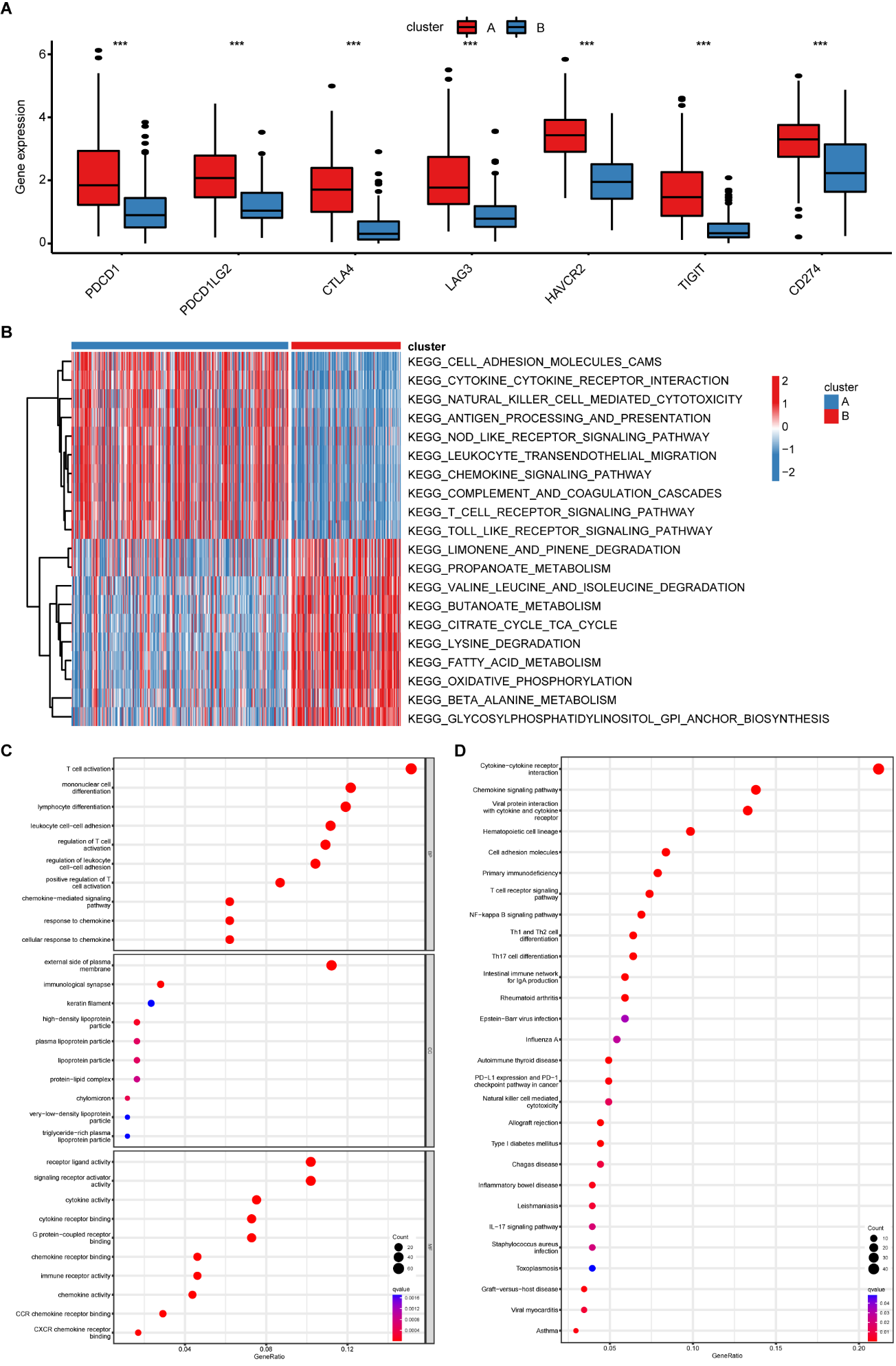


**Supplementary Figure S4.** The transcriptome features of the consensus clustering. **(A)** Comparison of immune checkpoint genes expression in the two clusters. **(B)** Heatmap displayed the activities of KEGG pathways in each cluster calculated by the GSVA algorithm. **(C), (D)** The results of GO and KEGG pathway analyses based on the DEGs of the two clusters.


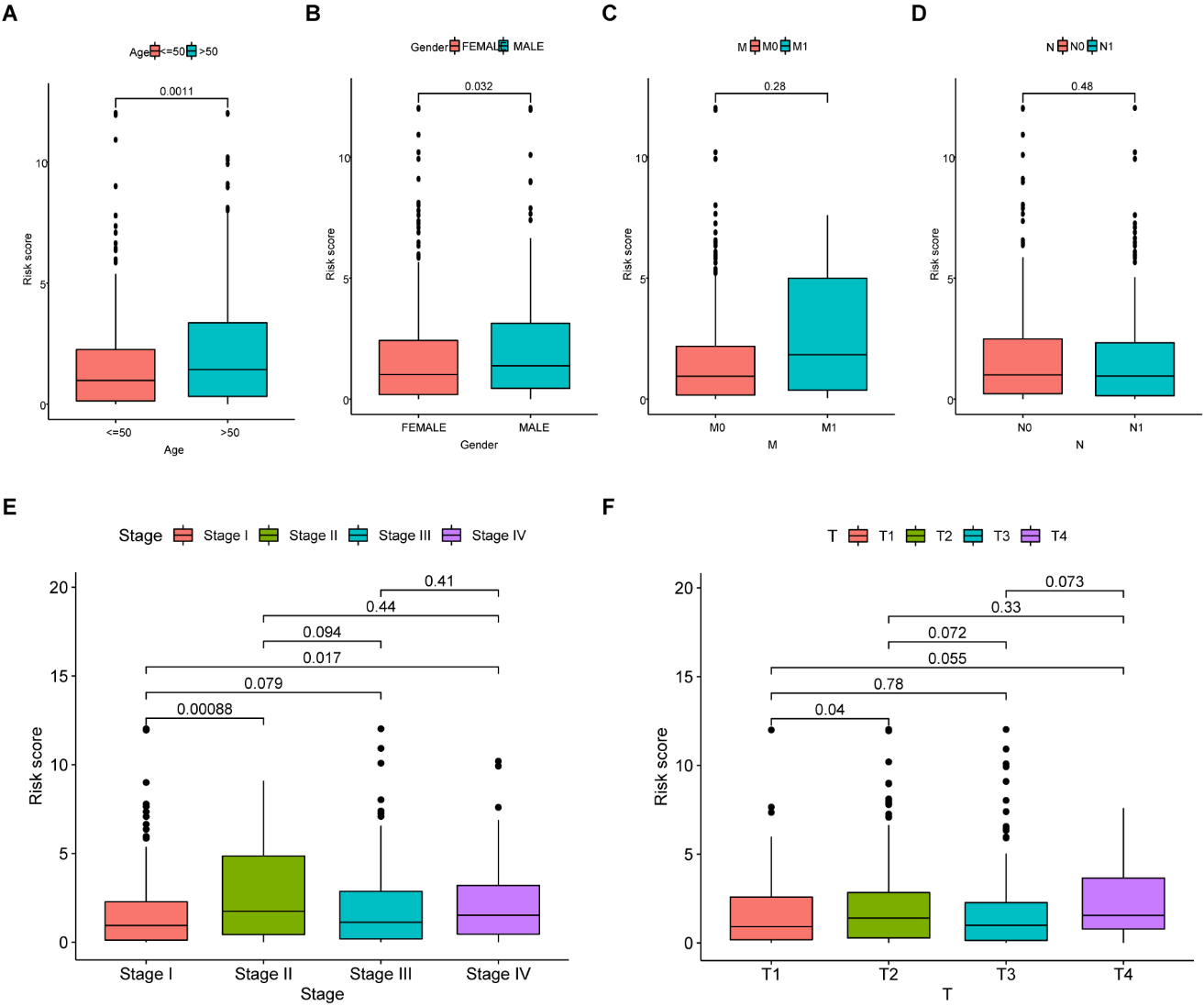


**Supplementary Figure S5.** The relationship between the risk score and the clinical characteristics. **(A), (B)** Patients older than 50 or male had a higher risk score. **(C), (D), (E), (F)** The relationship between the risk score and the TNM stage.


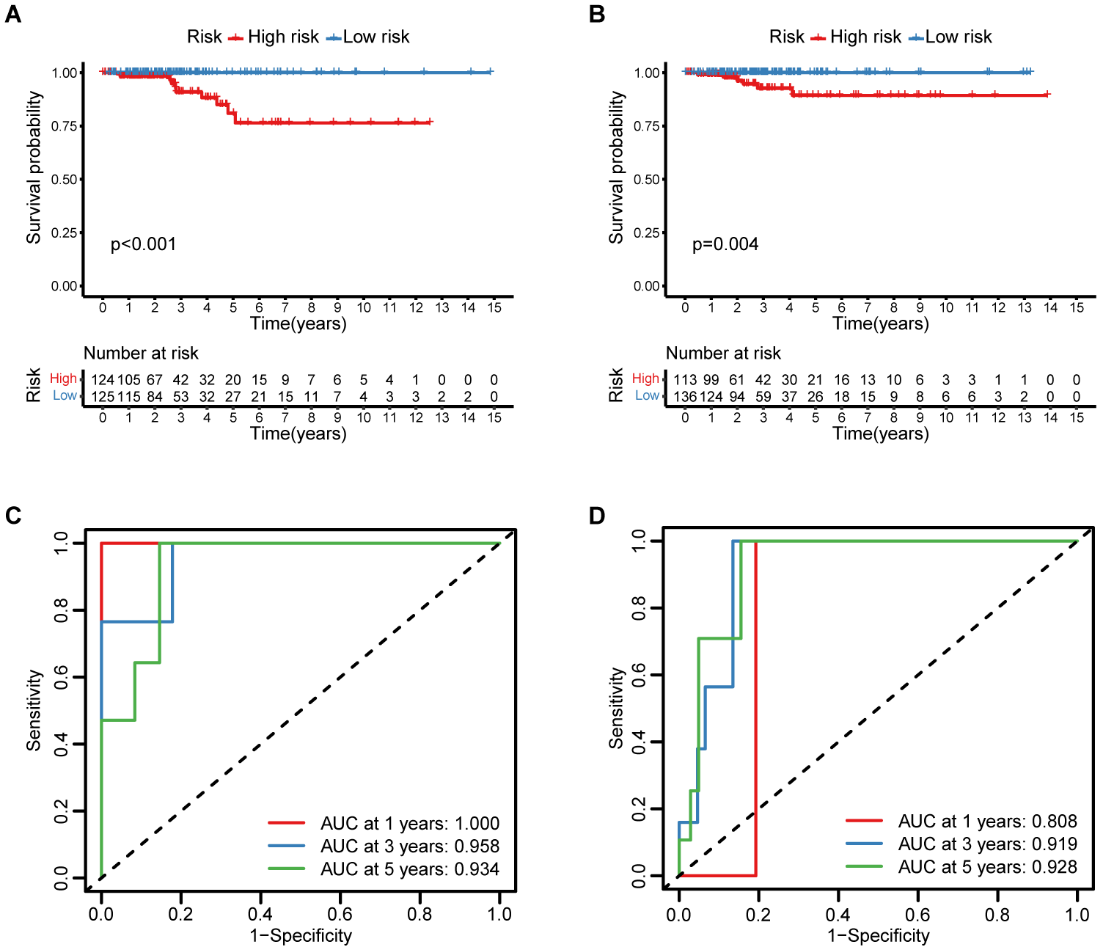


**Supplementary Figure S6.** The validation of the eight-gene prognostic signature. **(A), (B)** The Kaplan-Meier survival curve and the log-rank test showed different prognoses between the high-risk and low-risk groups in the train and test sets. **(C), (D)** The ROC curve displayed the accuracy of the prediction of 1-, 3-, 5- year survival states in the train and test sets.


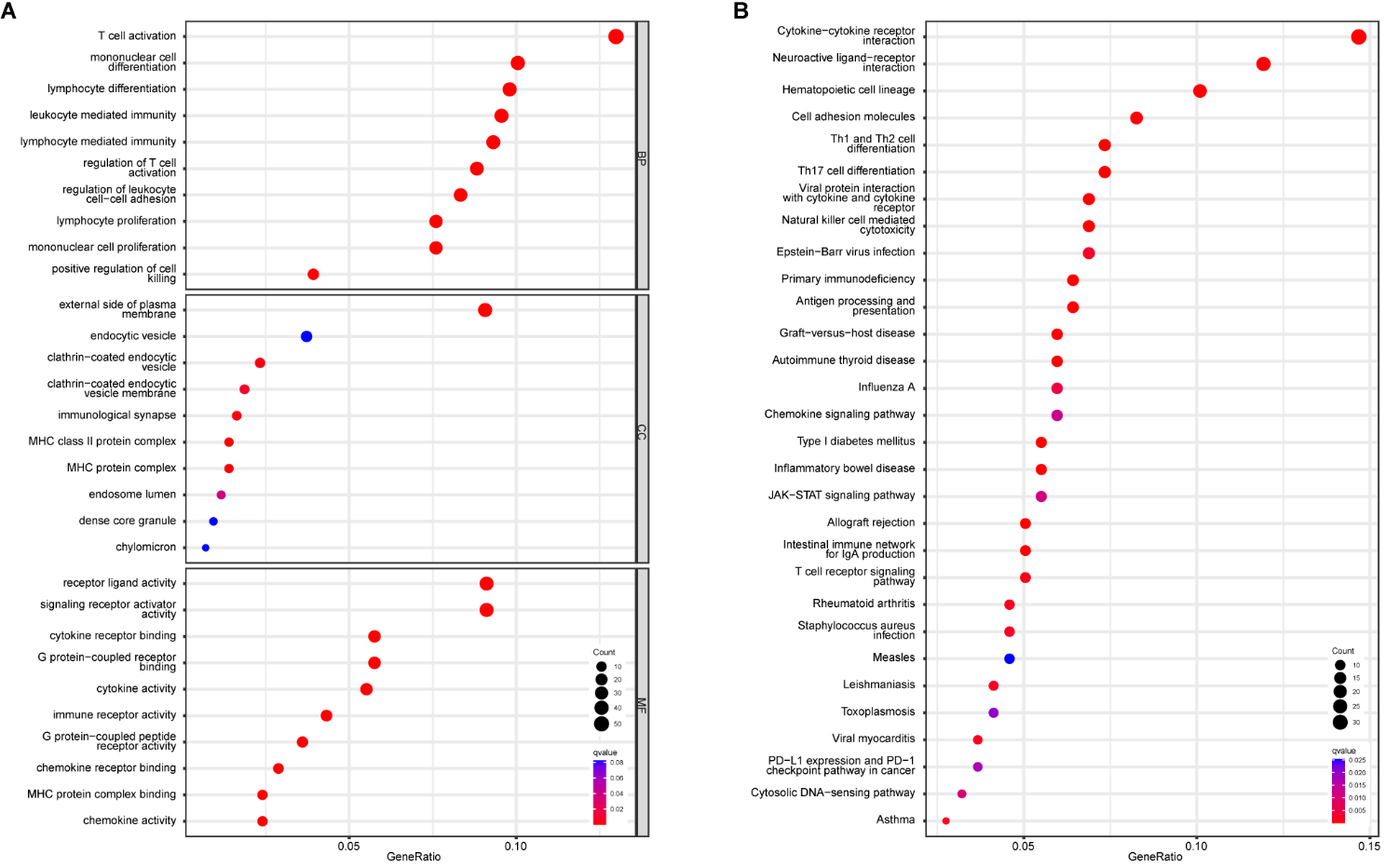


**Supplementary Figure S7.** The functional enrichment analysis of the risk group. **(A)** The GO analysis showed the significant biological process, cellular component, and molecular function between the high-risk and low-risk groups. **(B)** The enrichment of KEGG pathways based on the DEGs of the high-risk and low-risk groups.


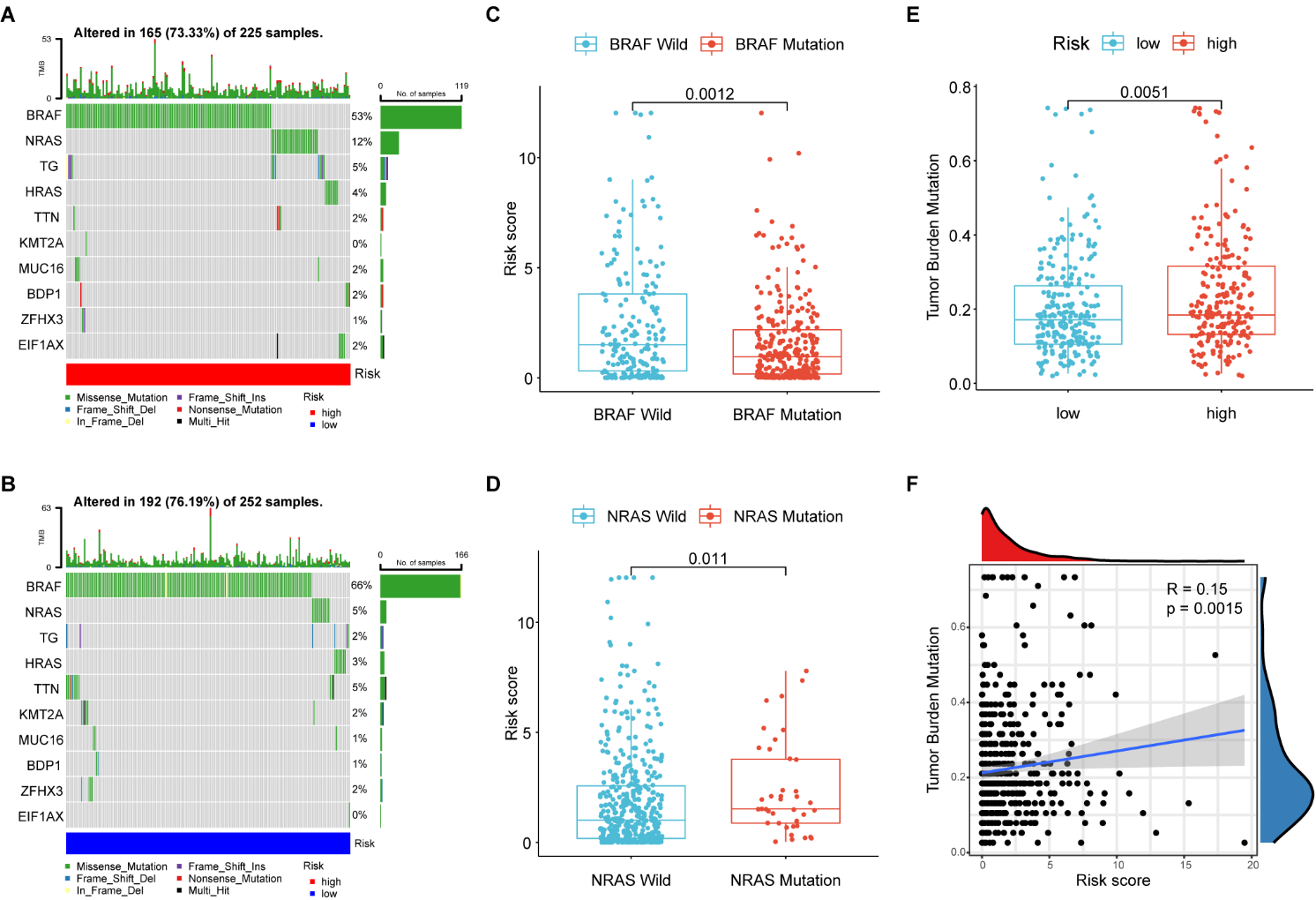


**Supplementary Figure S8.** The tumor mutation profiles of the high-ERGS and low-ERGS groups. (**A**), (**B**) The top 10 genes with the highest frequency of mutations in each ERGS group. (**C**), (**D**) The relationship between BRAF or NRAS mutation and the ERGS. (**E**), (**F**) The high-ERGS group had a higher TMB, which positively correlated with the ERGS.


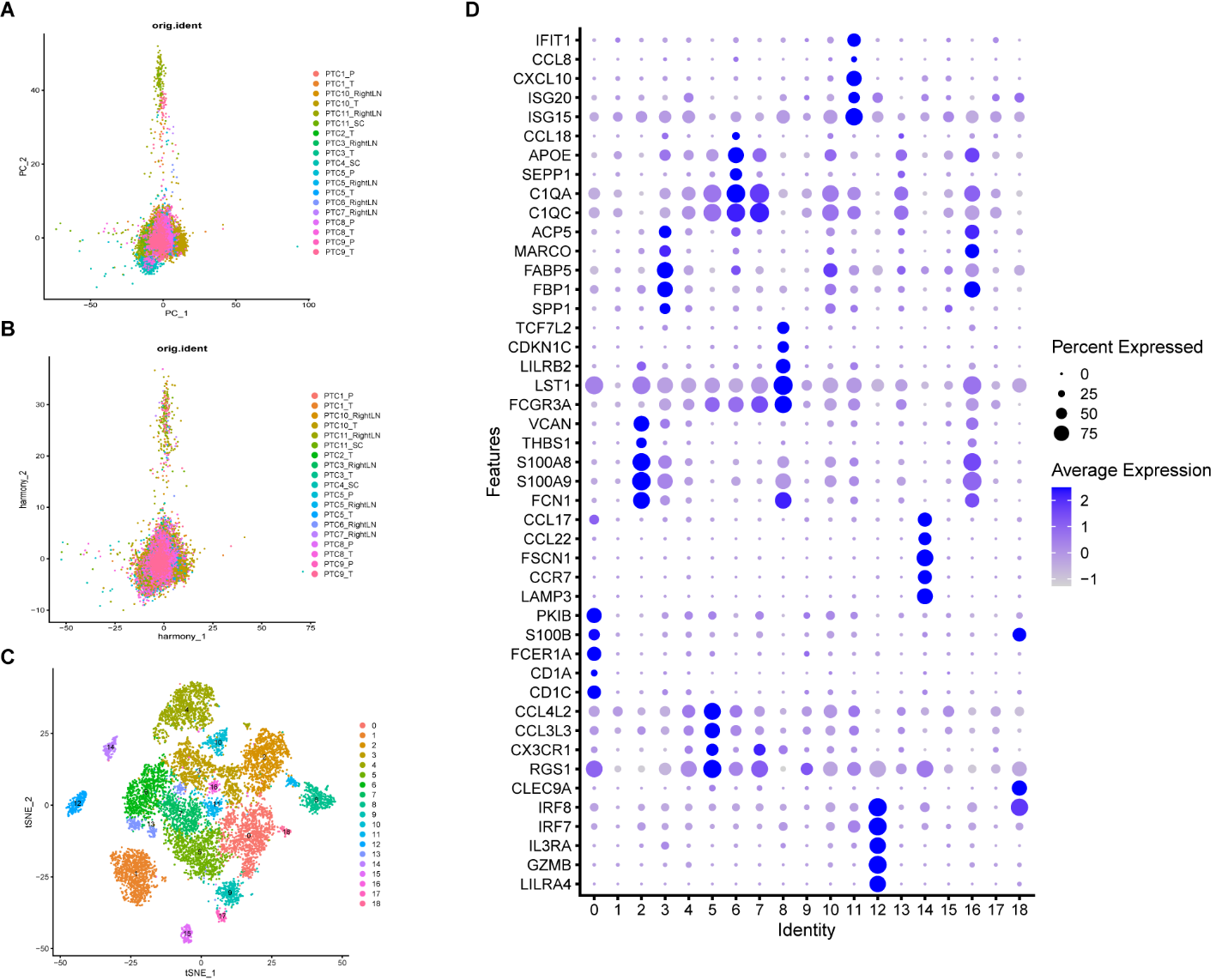


**Supplementary Figure S9.** The identification of the myeloid cell subtypes. **(A), (B)** The batch effect before and after the Harmony correction. **(C)** t-SNE plot of 18 subclusters after the "Seurat" pipeline. **(D)** Dot plot of the expression of the marker genes in the 18 subclusters.


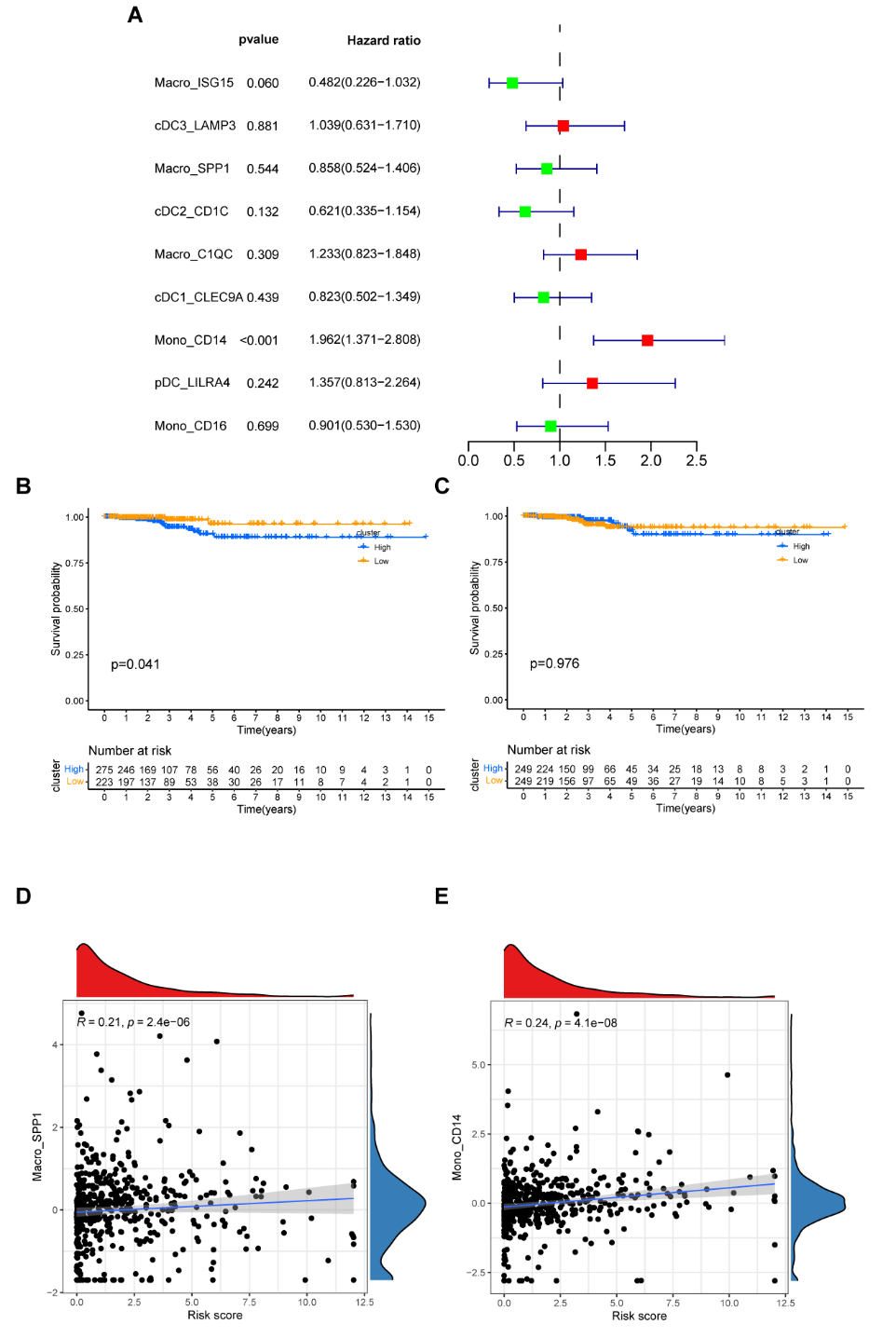


**Supplementary Figure S10.** The correlation analysis between the risk score and the infiltration of myeloid cell subtypes. **(A)** Univariate analysis showed that CD14^+^ monocyte infiltration could be a risk factor for poor prognosis. (**B**), (**C**) Survival analysis revealed the poor prognosis of CD14^+^ monocyte infiltration. **(D), (E)** The linear correlation analysis between the risk score and the cell infiltration.


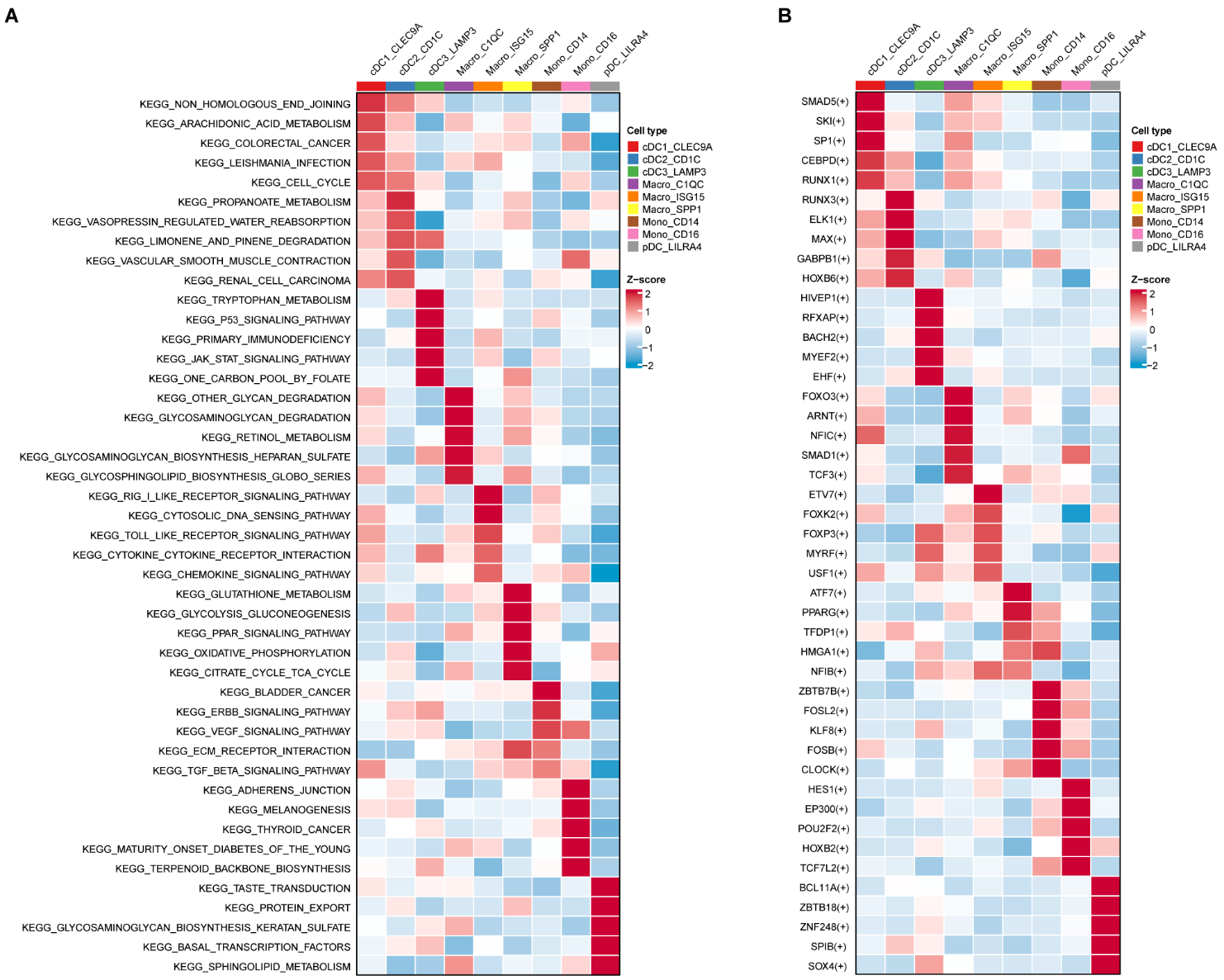


**Supplementary Figure S11.** The transcriptome characteristics of the CD14+ monocyte and SPP1+ macrophage. (**A**) Heatmap showed the highly upregulated KEGG pathways in each myeloid cell subtype using the GSVA algorithm. (**B**) Heatmap displayed the top 5 TFs with the highest activities in each myeloid cell subtype using the SCENIC analysis.


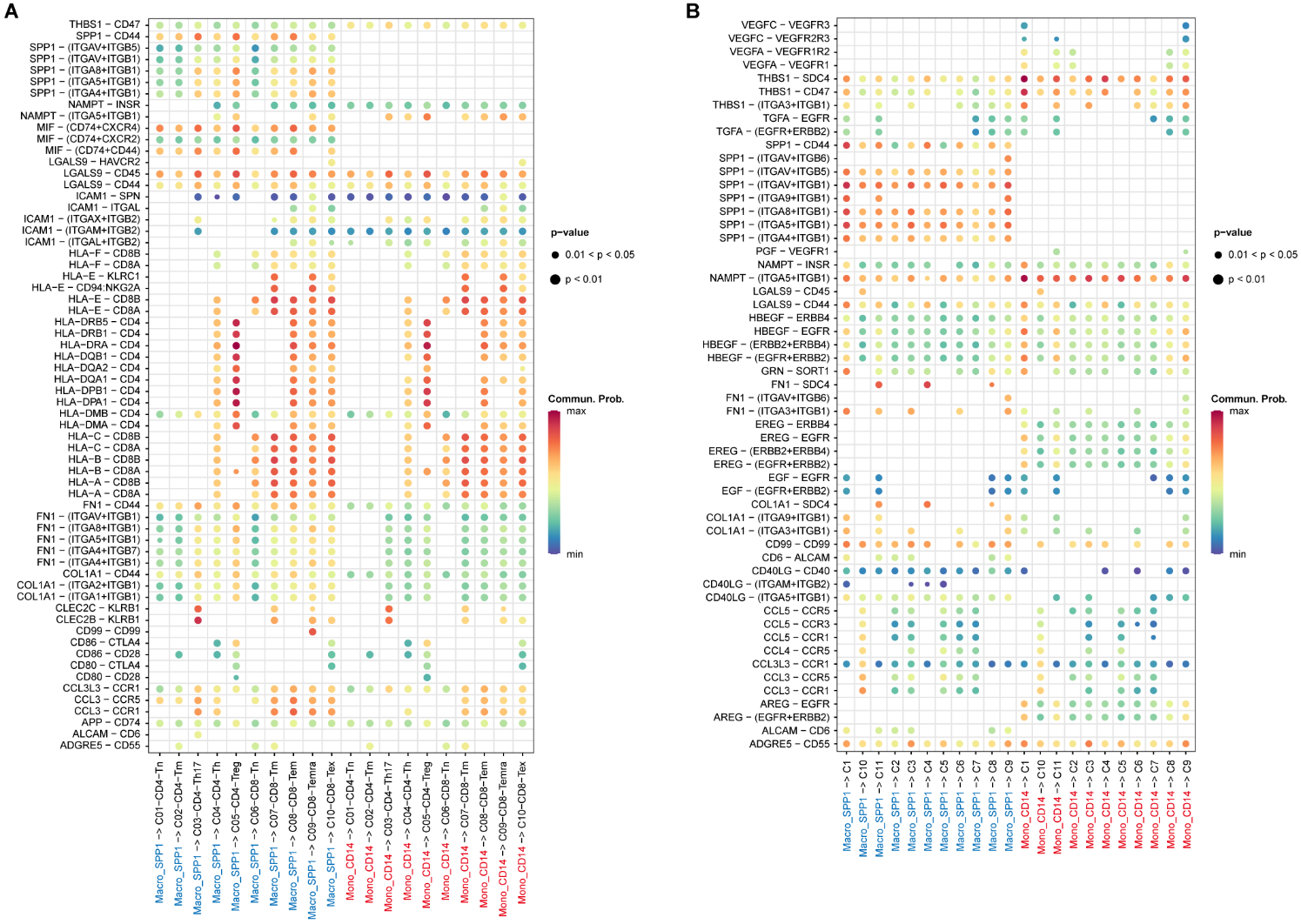


**Supplementary Figure S12.** The communication landscape of SPP1^+^ macrophage and CD14^+^ monocyte. **(A)** The landscape of SPP1^+^ macrophage and CD14^+^ monocyte crosstalk with T cell. **(B)** The landscape of SPP1^+^ macrophage and CD14^+^ monocyte crosstalk with tumor cell.
